# Cancer Cell Identification via Lysosomal Membrane Microviscosities Using a Green-Emitting BODIPY Molecular Rotor

**DOI:** 10.1021/jacsau.5c00253

**Published:** 2025-04-14

**Authors:** Ru̅ta Bagdonaitė, Rokas Žvirblis, Jelena Dodonova-Vaitku̅nienė, Artu̅ras Polita

**Affiliations:** † Department of Biospectroscopy and Bioelectrochemistry, Institute of Biochemistry, Life Sciences Center, Vilnius University, Saulėtekio av. 7, Vilnius LT-10257, Lithuania; ‡ Department of Biothermodynamics and drug design, Institute of Biotechnology, Life Sciences Center, Vilnius University, Saulėtekio av. 7, Vilnius LT-10257, Lithuania; § Department of Organic Chemistry, Faculty of Chemistry and Geosciences, Institute of Chemistry, Vilnius University, Naugarduko st. 24, Vilnius LT-03225, Lithuania; ∥ Department of Organic Chemistry, Center for Physical Sciences and Technology, Saulėtekio av. 3, Vilnius LT-10257, Lithuania

**Keywords:** lysosomes, microviscosity, lipids, BODIPY, FLIM, cancer

## Abstract

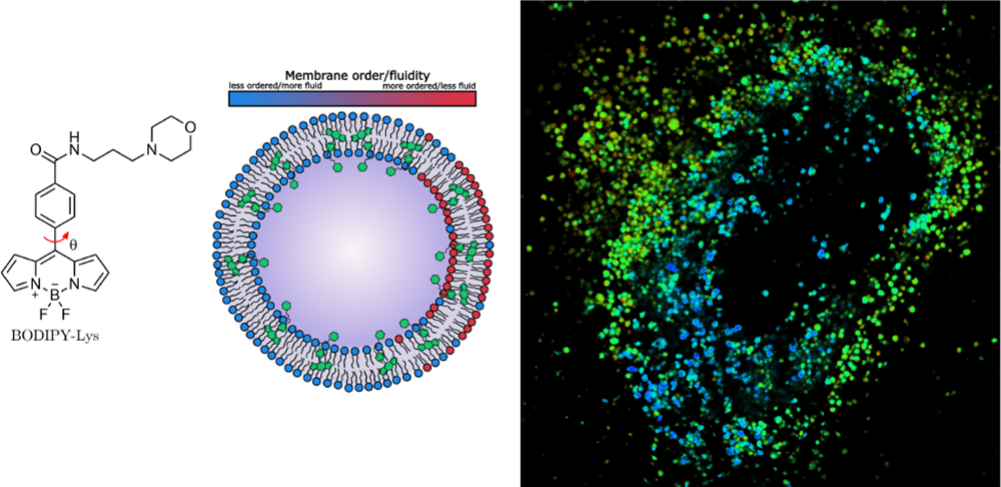

Lysosomes are dynamic, membrane-bound organelles that play key roles in cellular waste disposal, macromolecule recycling, and signaling. Disruptions in lysosomal function and lipid composition are implicated in a wide range of diseases including lysosomal storage disorders, fatty liver disease, atherosclerosis, and cancer. Imaging of the lysosomal lipid composition has the potential to not only enhance the understanding of lysosome-related diseases and their progression but also help identify them. In this work, we present a novel viscosity-sensitive, green-emitting BODIPY probe that can distinguish between ordered and disordered lipid phases and selectively internalize into the lysosomal membranes of live cells. Through the use of fluorescence lifetime imaging microscopy, we demonstrate that lysosomal membranes in multiple cancer cells exhibit significantly higher microviscosities compared to noncancer cells. The differences in lysosomal microviscosities provide an effective approach for identifying cancer cells and indicate that malignant cells may possess more oxidized and saturated lysosomal lipid membranes. Furthermore, we demonstrate the utility of viscosity-sensitive probes in quantifying the compositional changes in lysosomal membranes by investigating the effects of lysosome-permeabilizing cationic amphiphilic drugs (CADs), sertraline, and astemizole. Our results reveal that despite their functional similarities, these CADs exert opposite effects on lysosomal microviscosities in both cancerous and noncancerous cells, suggesting that different mechanisms may contribute to the CAD-induced lysosomal damage and leakage.

## 1 Introduction

Since their discovery, lysosomes have been predominantly associated with the waste disposal and recycling of macromolecules.^1^ Today, however, lysosomes are recognized as versatile signaling organelles with a variety of cellular functions that are required not only for normal cell survival^1^ but also for malignant transformation and cancer progression.^2,3^ Lysosomes not only provide cancer cells with energy and building blocks but also directly contribute to several common cancer traits, such as growth signaling, metastasis, angiogenesis, cell division, and drug resistance.^4^ To meet the altered metabolic and survival requirements brought about by malignant transformation, lysosomes undergo morphological and compositional changes during cancer progression.^5,6^ Most aggressive cancers feature enlarged lysosomal volume and more peripheral localization of the lysosomes.^6,7^ Furthermore, malignant transformations compromise the stability of lysosomal membranes due to various metabolic and signaling abnormalities that increase the production of ROS, which in turn oxidize and destabilize lysosomal membrane lipids.^8^ Since oxidized lipids feature a greater number of hydrogen bonds in the hydrophobic core of the bilayer, such lipid bilayers are more tightly packed and hence more ordered.^9−11^ Additionally, malignant cells typically contain elevated quantities of saturated lipids,^12,13^ which often increase the lipid order of biological membranes.^14^ In addition to cancer, changes in lysosomal lipid composition and the accumulation of cholesterol within lysosomes are also observed in conditions like atherosclerosis,^15^ lysosomal storage diseases,^16^ and fatty liver disease.^17^ Consequently, the ability to assess lysosomal membrane lipid order could not only aid in identifying cancer cells and provide insights into tumor aggressiveness but also offer valuable information for research into atherosclerosis, lysosomal storage diseases, and fatty liver disease. Additionally, emerging evidence suggests that cancer cells frequently acquire mutations that allow them to evade therapy-induced apoptosis while becoming vulnerable to lysosome-dependent cell death, which proceeds upon permeabilization of lysosomal membranes.^6^ Notably, lysosome-dependent cell death operates independently of caspase activation and may be particularly effective in treating apoptosis-resistant cancers.^6^ A number of clinically approved cationic amphiphilic drugs (CADs), commonly used to treat neurological disorders, have been shown to induce lysosomal membrane permeabilization and trigger lysosomal-dependent cell death.^18^ Lysosomal membrane permeabilization here refers to the release of lumenal contents into the cytosol, potentially leading to lysosome-dependent cell death.^19^ CADs typically contain one or more amine groups that can be protonated at physiological pH, causing them to accumulate within lysosomes.^20^ This accumulation disrupts the cancer-promoting and cancer-supporting roles of lysosomes^21,22^ and destabilizes lysosomal membranes,^18,21,22^ opening promising possibilities for treating chemotherapy-resistant cancers. While numerous studies have explored the inhibitory effects of CADs on various enzymes involved in lysosomal membrane permeabilization,^18,20,21^ the precise impact of CADs on the lipid order of lysosomal membranes, which likely affects membrane permeabilization, remains unclear. Furthermore, although structurally different CADs demonstrate similar effects on lysosomal permeabilization and protein inhibition,^23^ it is still unknown whether different CADs have comparable or distinct effects on the lipid packaging characteristics of lysosomal membranes.

Microviscosity measurements provide one of the most convenient ways to track compositional changes in lysosomal membranes as the microviscosity values are highly influenced by lipid packing efficiency and lipid order.^10,24,25^ For instance, cholesterol-rich bilayers form highly viscous, liquid-ordered lipid phases, while highly unsaturated lipids produce nonviscous and liquid-disordered lipid phases.^26,27^ The term microviscosity here refers to the lipid packaging and molecular mobility of the probe in the local environment. Viscosity-sensitive dyes, known as molecular rotors, can quantify microviscosity changes produced by cholesterol variations or lipid phase separations, making them valuable for studying compositional changes in lipid systems.^28−30^ In the excited state, molecular rotors can undergo intramolecular rotation, resulting in a molecular rotor entering the dark state.^30,31^ Thus, in low-viscosity environments or disordered lipid bilayers, the intramolecular rotation of the rotor is not hindered, and nonradiative decay dominates, resulting in a decrease in the fluorescence quantum yield and lifetime.^32^ In contrast, in high-viscosity environments or ordered lipid bilayers, the intramolecular rotation is inhibited, leading to increased fluorescence lifetimes.^32^ When paired with fluorescence lifetime imaging microscopy (FLIM), molecular rotors can produce spatial microviscosity maps of lipid structures, revealing dynamic changes in the lipid bilayer composition.^33−36^ Many successful BODIPY-based lysosome-targeting viscosity probes, such as Lyso-V^37^ and Lyso-B,^38^ incorporate an electron-rich morpholine group near the BODIPY core. This design causes fluorescence quenching via photoinduced electron transfer (PET) at neutral or basic pH levels. While PET effectively ensures that the probe is fluorescent only in acidic lysosomes, it limits the detection of lysosomes in pathological conditions, where elevated pH levels are common.^39,40^ Moreover, the morpholine’s proximity to the BODIPY core in probes such as Lyso-V interferes with the molecular rotor’s viscosity-sensing mechanism, reducing its sensitivity to viscosity changes.^37^

The aim of this research is to explore the potential for identifying cancer and noncancer cells based on lysosomal membrane microviscosity and to demonstrate the broad applicability of microviscosity measurements in evaluating the effects of lysosome-targeting chemotherapeutic drugs. To the best of our knowledge, no studies to date have investigated the possibility of using lysosomal membrane fluidity as a biomarker for cancer detection. In this work, we introduce BODIPY-Lys, a BODIPY-C_10_ derivative with high specificity for lysosomes and a fluorescence lifetime-based ability to measure the microviscosity of lysosomal membranes. We explore the photophysical properties of BODIPY-Lys and demonstrate that the fluorescence lifetime of BODIPY-Lys is mainly affected by the viscosity and not the polarity or temperature of the environment. Using BODIPY-Lys in combination with FLIM, we image lysosomal microviscosities in four distinct human cancerous and noncancerous cell lines. Our results reveal that lysosomal membrane microviscosities in cancerous cells are about three times higher compared to noncancerous cells, indicating that the biophysical properties of lysosomal membranes could serve as a biomarker for malignancy detection. Finally, we assess the effects of two commonly used CADs, sertraline (Ser) and astemizole (Ast), on lysosomal membrane microviscosities in both cancerous and noncancerous cell lines. Although Ser and Ast share similar mechanisms for inducing lysosomal membrane permeabilization, microviscosity measurements show that both CADs have opposite effects on lipid bilayer fluidity in cancerous cell lines.

## 2 Results and Discussion

### 2.1 Probe Design

**Figure 1:**
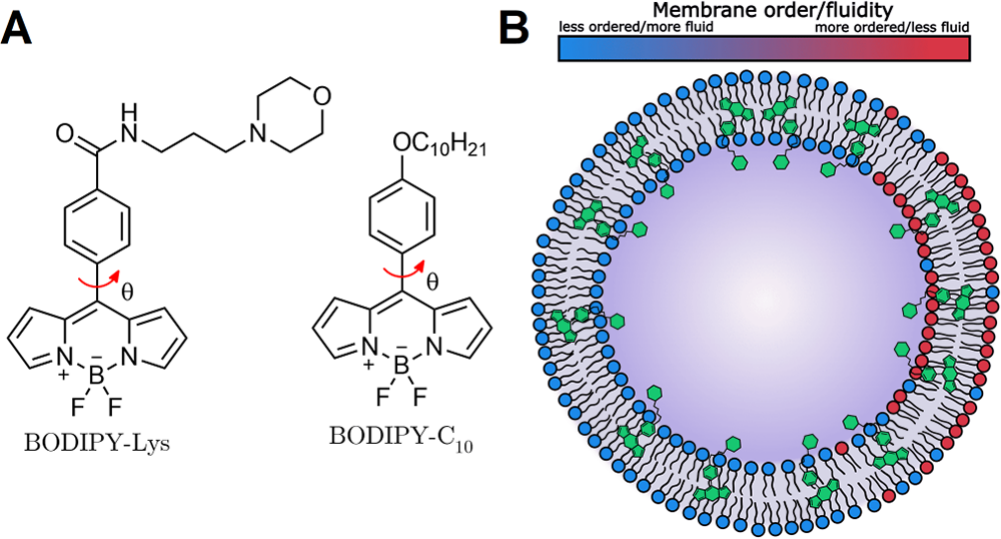
(A) Structures of one of the most widely used molecular rotors, BODIPY-C10, and its variant for lysosomes, BODIPY-Lys. Red arrows indicate intramolecular rotation, which causes BODIPY to display viscosity sensitivity. (B) Supposed positioning of BODIPY-Lys in the lysosomal membrane.

The design of BODIPY-Lys is based on one of the most widely used and successful molecular rotors to date, BODIPY-C_10_. To retain the viscosity-sensitive mechanism of BODIPY-C_10_, we preserved the rotation of the phenyl group relative to the BODIPY core unhindered and modified the para-position of the phenyl group by introducing a morpholine group (Figure 1A). The morpholine group provides BODIPY-Lys with reasonable water solubility and becomes protonated only in the acidic environment of the lysosomes, leading to the gradual accumulation of BODIPY-Lys in these organelles. The distant placement of the morpholine group from the BODIPY core prevents PET from occurring and enables the detection of lysosomes with elevated pH levels. The hydrophobic core of BODIPY, along with the phenyl ring, likely integrates into the lysosomal lipid membrane, while the protonated morpholine group keeps the probe anchored to the inner leaflet of the lipid bilayer (Figure 1B). The synthesis of BODIPY-Lys is described in the electronic Supporting Information.

### 2.2 Absorption, Steady-State, and Time-Resolved Fluorescence

**Figure 2:**
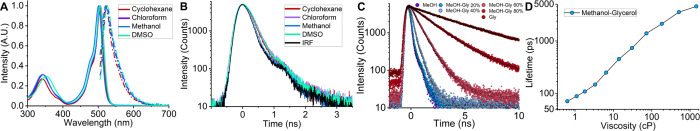
Photophysical characterization of BODIPY-Lys. (A) Fluorescence (dotted-dashed lines) and absorption (solid lines) spectra of BODIPY-Lys obtained in cyclohexane, chloroform, DMSO, and methanol. (B) Time-resolved fluorescence decays of BODIPY-Lys in cyclohexane, chloroform, DMSO, and methanol. (C) Time-resolved fluorescence decays of BODIPY-Lys obtained in methanol–glycerol mixtures of varying viscosities. (D) Fluorescence lifetimes of BODIPY-Lys in methanol–glycerol mixtures.

To investigate the photophysical characteristics of BODIPY-Lys, we performed absorption, steady-state, and time-resolved fluorescence measurements in solvents with varying polarities, from nonpolar cyclohexane to polar DMSO (Figure 2). Since lysosomal membranes contain substantial amounts of proteins and carbohydrates,^41^ a lipid order probe must remain insensitive to environmental polarity to accurately measure the microviscosity of the protein-rich lipid bilayer. Similar to BODIPY-C_10_,^31^ BODIPY-Lys exhibits two absorption bands: a low-intensity band in the 300–400 nm region and a main band in the 450–525 nm region (Figure 2A). The fluorescence spectra of BODIPY-Lys display a minor red shift as the solvent polarity increases, with the fluorescence peak maximum shifting from 522 nm in cyclohexane to 525 nm in DMSO (Figure 2A). The absence of red-shifted bands in the fluorescence spectra demonstrates that BODIPY-Lys does not form aggregates or dimers in either polar or nonpolar solvents, unlike many other BODIPY dyes.^42^

To assess how solvent polarity influences the fluorescence lifetimes of BODIPY-Lys, we performed time-resolved fluorescence measurements in cyclohexane, toluene, chloroform, dichloromethane, methanol, and DMSO (Figures 2B and S1). BODIPY-Lys exhibited monoexponential fluorescence decays in all solvents, with lifetimes ranging from 126 ps in cyclohexane to 138 ps in DMSO. The correlation between the solvent polarity and fluorescence lifetimes was marginal, with only slight variations across solvents. For instance, BODIPY-Lys displayed lifetimes of 71 ps in methanol (0.63 cP), 116 ps in dichloromethane (0.43 cP), and 151 ps in chloroform (0.56 cP), indicating minimal dependence on solvent polarity (Figure S1). In contrast, the fluorescence lifetimes of BODIPY-C_10_ range from 300 to 800 ps in low-viscosity solvents of varying polarities.^31^

To evaluate the viscosity sensitivity of BODIPY-Lys and construct a fluorescence lifetime-viscosity calibration curve, we performed time-resolved and steady-state fluorescence measurements of BODIPY-Lys in methanol–glycerol mixtures spanning a viscosity range from 0.6 to 1457 cP (Figures 2C and S2). This calibration curve enables the conversion of BODIPY-Lys fluorescence lifetimes in lysosomal membranes into universal viscosity values, facilitating comparisons to other microviscosity measurements in various organelles. BODIPY-Lys displays excellent viscosity sensitivity, with fluorescence lifetimes ranging from 71 ps in methanol (0.6 cP) to 4545 ps in pure glycerol (1457 cP) (Figure 2C,D). The fluorescence decays of BODIPY-Lys were monoexponential in methanol/glycerol mixtures up to 50% glycerol. Beyond 60% glycerol, BODIPY-Lys exhibited biexponential decays with a low-amplitude, short-lifetime component (Figure S2). Furthermore, BODIPY-Lys displays a broader dynamic viscosity range than BODIPY-C10, owing to its lower fluorescence lifetime values in nonviscous solvents. The dynamic viscosity range, which can be calculated from the fluorescence lifetime ratio at high and low viscosities, is 64.0 for BODIPY-Lys in methanol–glycerol mixtures, compared to 11.2 for BODIPY-C10 in the same mixture.^31^

To assess whether the protonation of the morpholine group affects the photophysical properties of BODIPY-Lys—an important factor at the acidic pH of lysosomes—we conducted steady-state and time-resolved fluorescence measurements in methanol, ethanol, and isopropyl alcohol with added sulfuric acid (Figure S3). The protonation of morpholine did not significantly affect the fluorescence lifetimes or intensities, with changes in fluorescence lifetimes limited to only a few picoseconds following the addition of sulfuric acid (Figure S3).

Finally, since BODIPY dyes occasionally display temperature sensitivity,^43^ which could interfere with their use at physiological temperatures, we quantitatively assessed BODIPY-Lys’s temperature sensitivity by conducting temperature-dependent fluorescence decay measurements in DMSO (Figure S4). The temperature sensitivity was evaluated using the relative sensitivity parameter *S* over a temperature range of 20–70 °C (eq 1).^44^
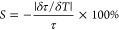
1

The expression for *S* represents the percentage change in lifetime per degree of temperature change, where τ is the fluorescence lifetime and δ*T* is the temperature change in degrees Celsius (°C). The fluorescence lifetimes of BODIPY-Lys showed minimal temperature dependence with a relative sensitivity *S* value of just 0.15%/°C (Figure S4). In summary, our findings confirm that the fluorescence lifetimes of BODIPY-Lys are mainly influenced by solvent viscosity, with only negligible effects from solvent polarity and temperature. This makes BODIPY-Lys a reliable probe for microviscosity measurements in biological, protein-rich environments, even at physiological temperatures.

Given that cholesterol is distributed heterogeneously in biological membranes and lipid bilayers contain ordered nanodomains,^45^ it is essential for a membrane probe to partition into both liquid-ordered (Lo) and liquid-disordered (Ld) lipid phases while accurately assessing their microviscosity. In this context, we aimed to evaluate BODIPY-Lys’s ability to differentiate between Lo and Ld phases based on their microviscosity and to examine the probe’s response to varying cholesterol concentrations in the Ld phase. To achieve this, we performed FLIM on BODIPY-Lys in giant unilamellar vesicles (GUVs) composed of Ld (DOPC), Lo (DOPC/DPPC/Chol), and intermediate Ld-Lo (DOPC/Chol) lipid phases.

**Figure 3:**
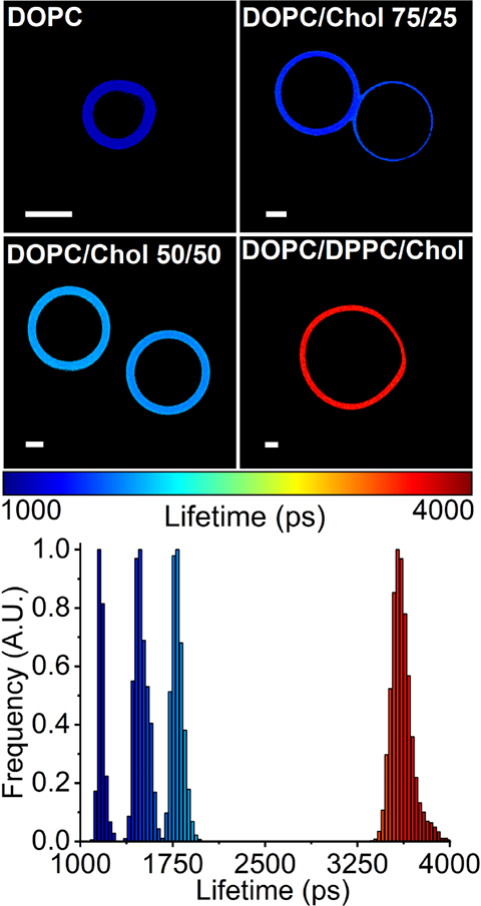
FLIM of BODIPY-Lys in DOPC, DOPC/Chol (75/25), DOPC/Chol (50/50), and DOPC/DPPC/Chol (1:5:5) GUVs. The corresponding BODIPY-Lys fluorescence lifetime histogram is shown in the bottom panel. Scale bars are 2 μm.

BODIPY-Lys successfully stained both Lo and Ld lipid phases at a dye-to-lipid ratio of 1:800, displaying intensity-weighted fluorescence lifetimes of approximately 1160 ps in the Ld (DOPC) phase and 3610 ps in the Lo (DOPC/DPPC/Chol) phase (Figures 3 and S5). These fluorescence lifetimes correspond to viscosities of 55 cP for DOPC and 520 cP for DOPC/DPPC/Chol and are in good agreement with both theoretically and experimentally determined values of approximately 50 cP for DOPC^46,47^ and experimentally measured values of 413 ± 128 cP for the Lo phase of DOPC/DPPC/Chol.^48^ By integrating into highly ordered lipid phases and displaying significant fluorescence lifetime differences between Lo and Ld phases, BODIPY-Lys can distinguish lipid phases based on their microviscosity. This capability is particularly valuable for detecting highly ordered lipid domains in lysosomal membranes.^49^

The addition of cholesterol to Ld (DOPC) GUVs led to a significant increase in the intensity-weighted fluorescence lifetimes of BODIPY-Lys, rising from 1160 ps in pure DOPC GUVs to 1490 ps (80 cP) in DOPC/Chol (75/25) GUVs and 1790 ps (130 cP) in DOPC/Chol (50/50) GUVs (Figure 3). This result is consistent with cholesterol’s role in organizing and condensing Ld lipid bilayers.^50^ The biexponential nature of BODIPY-Lys fluorescence decays in GUVs likely arises from the probe adopting multiple orientations within the lipid bilayer. Similarly, the transition from monoexponential decays in solvents to multiexponential decays in lipid membranes has been attributed to the adoption of multiple orientations in other BODIPY derivatives.^51,52^

Importantly, BODIPY-Lys’s ability to distinguish between Lo and Ld lipid phases and accurately reflect cholesterol-induced changes in lipid order can be used to estimate cholesterol and unsaturated lipid concentrations in lysosomes as well as to study biophysical changes in lysosomal membranes upon protein binding and to image the ordering and disordering effects of small molecules.

### 2.3 FLIM of BODIPY-Lys in Live Human Cancerous and Noncancerous Cells

**Figure 4:**
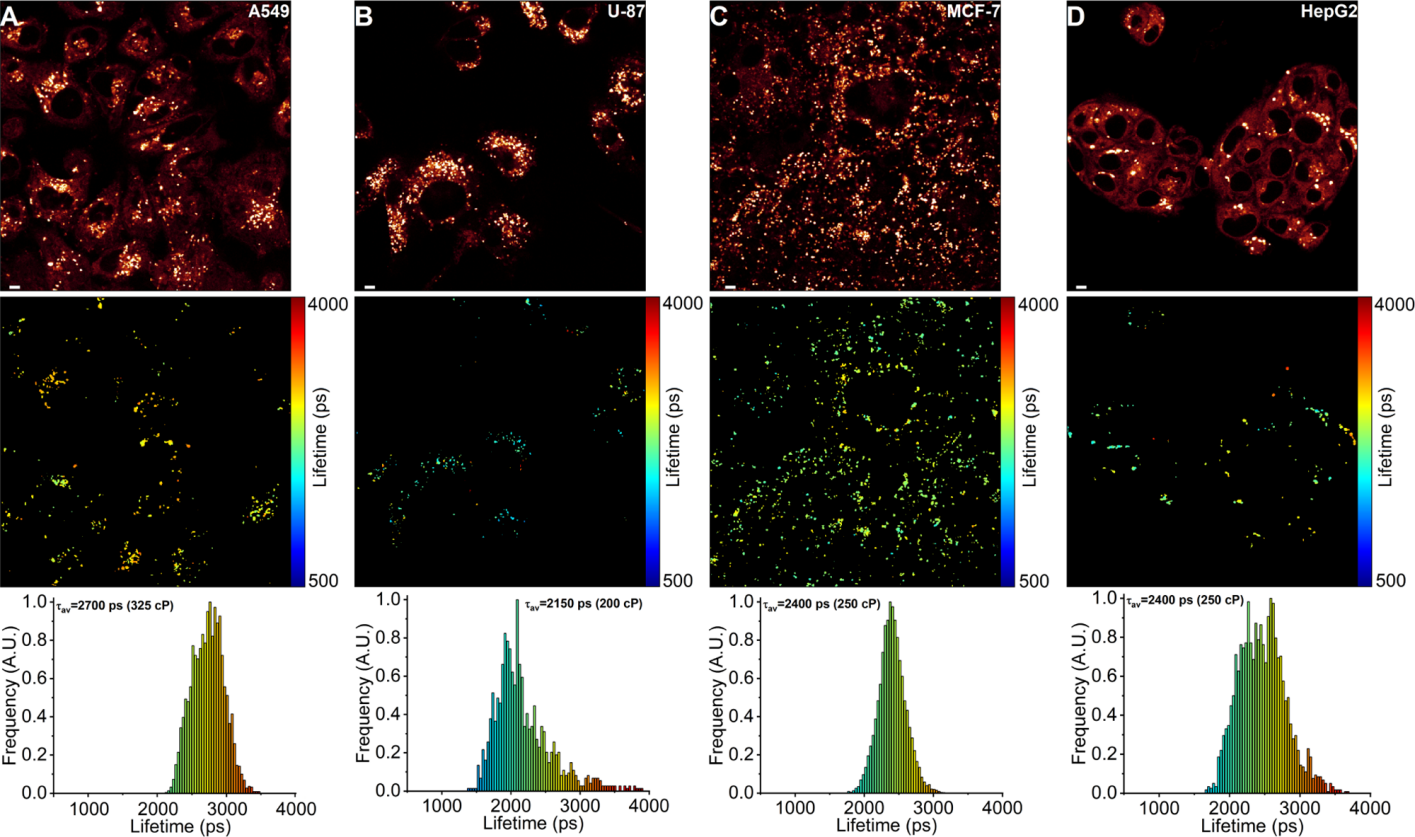
FLIM of BODIPY-Lys in human cancerous cell lines. (A) Human lung cancer A549. (B) Human glioblastoma U-87. (C) Human breast cancer MCF-7. (D) Human liver cancer HepG2. The top panel shows images of the fluorescence intensity. FLIM images are shown in the middle panel. The corresponding lifetime histograms with mean fluorescence lifetimes τ_av_ and corresponding viscosities in the methanol–glycerol calibration mixtures are shown in the bottom panel. Scale bars are 5 μm.

**Figure 5:**
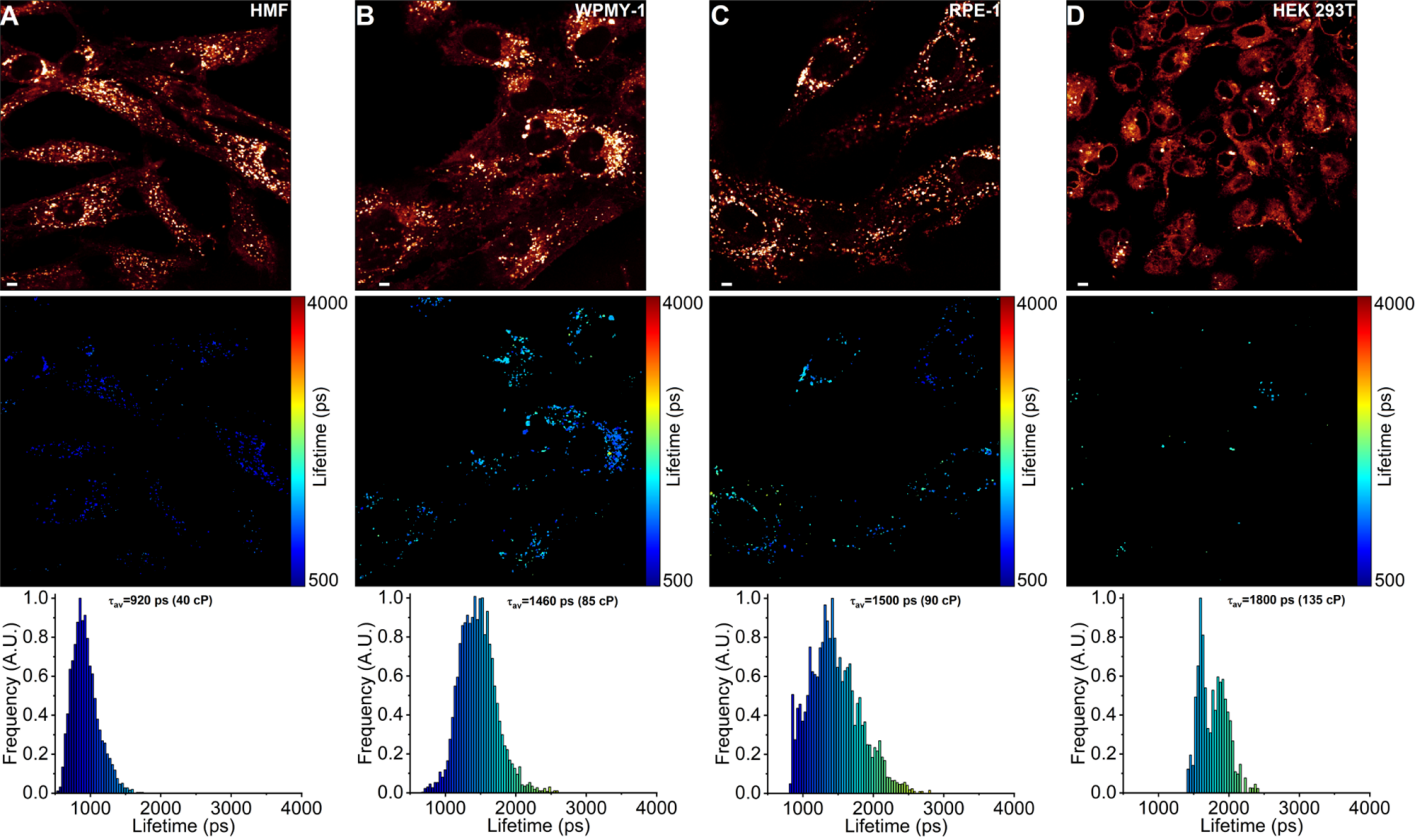
FLIM of BODIPY-Lys in human noncancerous cell lines. (A) HMFs. (B) Human prostate myofibroblasts WPMY-1. (C) Human retinal pigment epithelium cells RPE-1. (D) Human embryonic kidney cells HEK 293T. The top panel shows images of the fluorescence intensity. FLIM images are shown in the middle panel. The corresponding lifetime histograms with mean fluorescence lifetimes τ_*av*_ and corresponding viscosities in the methanol–glycerol calibration mixtures are shown in the bottom panel. Scale bars are 5 μm.

Next, we performed FLIM of BODIPY-Lys in four different human cancer lines: lung cancer (A549), glioblastoma (U-87), breast cancer (MCF-7), and liver cancer (HepG2). In all cases, BODIPY-Lys was added to the cell medium at 0.5 μM concentrations without washing, yielding excellent fluorescence intensities in the lysosomes, which were about 100 times higher compared to the cytoplasm (Figure 4). To verify the lysosomal localization of BODIPY-Lys, we performed fluorescence colocalization in HepG2 cells with fluorescent dye neutral red, a known lysosomal marker (Figure S6).^53^ BODIPY-Lys displayed excellent colocalization with neutral red and effectively labeled lysosomal membranes across a broad range of lipid orders, including both highly ordered and disordered lysosomes (Figures 4 and 5). Of note, no morphological changes were observed in live cells following BODIPY-Lys staining (Figure S7), indicating low probe toxicity, consistent with the generally low toxicity of BODIPY dyes.^54^ To ensure that the microviscosity measurements are accurate, we recorded the steady-state fluorescence spectra of BODIPY-Lys in lysosomes and confirmed the absence of red-shifted fluorescence bands, indicating that BODIPY-Lys does not aggregate in the lysosomal membranes and exhibits properties similar to those observed in organic solvents (Figure S8). FLIM analysis revealed that the fluorescence decays of BODIPY-Lys in lysosomal membranes were biexponential, suggesting that the probe adopts multiple orientations within the lipid bilayer, similar to its behavior in GUVs (Figures S8 and S9).

Interestingly, all investigated cancer cell lines contained moderately viscous and ordered lysosomes, with mean BODIPY-Lys intensity-weighted fluorescence lifetimes of 2730 ps (325 cP) in A549, 2150 ps (200 cP) in U-87, and 2400 ps (250 cP) in both MCF-7 and HepG2 cells (Figure 4). Notably, the number of lysosomes per cell varied significantly, with HepG2 cells having fewer but larger lysosomes compared to other cell lines. To compare lysosomal membrane microviscosities between cancerous and noncancerous cells, we performed FLIM of BODIPY-Lys in four noncancerous human cell lines: human mammary fibroblasts (HMFs), prostate myofibroblasts (WPMY-1), retinal pigment epithelium (RPE-1), and human embryonic kidney (HEK 293T) cells (Figure 5). BODIPY-Lys exhibited mean intensity-weighted fluorescence lifetimes of about 920 ps (40 cP) in HMF, 1460 ps (85 cP) in WPMY-1, 1500 ps (90 cP) in RPE-1, and 1800 ps (135 cP) in HEK 293T cells. In contrast to cancer cell lines, where lysosomal membrane microviscosities ranged from 200 to 325 cP, noncancerous cells exhibited significantly lower lysosomal microviscosities, ranging from 40 to 135 cP. The distinct differences in BODIPY-Lys fluorescence lifetimes between cancerous and noncancerous cells indicate that lysosomal membranes in cancer cells have a much higher lipid packing efficiency and are more ordered. Additionally, the lysosomal membranes in cancerous cells, particularly in U-87 and HepG2 (Figure 4B,D), exhibited significant heterogeneity in microviscosity, both between and within individual cells, indicating that the lipid composition of each lysosome varies significantly in cancer. In contrast, lysosomes in noncancerous cells displayed a lower degree of heterogeneity (Figure 5). Given that the fluorescence lifetime response of BODIPY-Lys to viscosity is nonlinear, differences between 1000 and 2000 ps, as seen in noncancerous RPE-1 cells, result in relatively modest changes in microviscosity, from 45 to 165 cP (Figure 5C). Meanwhile, even in a relatively homogeneous cancer cell line like MCF-7, BODIPY-Lys lifetime variations from 2000 to 3000 ps correspond to much greater differences in microviscosity, ranging from 165 to 390 cP (Figure 4C).

We hypothesize that lysosomal membranes in malignant cells likely contain a higher amount of saturated lipids, which often form highly viscous Lo lipid phases. In support of this hypothesis, various studies have shown that cancer cells overexpress fatty acid synthase, which increases the production of saturated fatty acids and leads to a more saturated lipidome.^12,13^ Additionally, cancer-specific signaling and metabolic abnormalities result in an increased reactive oxygen species (ROS) generation,^8^ which can oxidize membrane lipids, further increasing microviscosities due to enhanced hydrogen bonding in the lipid bilayer’s hydrophobic core. To confirm the presence of elevated ROS levels in our selected cell lines, we performed fluorescence intensity imaging using the commercially available ROS detection probe DCFH-DA.^55^ The fluorescence intensities of DCF, the ROS-oxidation product of DCFH-DA, were approximately 5–6 times higher in the cancerous cell lines HepG2 and U-87 compared to the noncancerous HMF and WPMY-1 cells, indicating significantly elevated ROS levels in malignant cells (Figure S10). Moreover, DCF fluorescence intensities were markedly uneven in cancerous cells, whereas noncancerous cell lines exhibited more uniform fluorescence intensities. This pattern closely resembled the highly heterogeneous lysosomal microviscosities observed in cancerous HepG2 and U-87 cells, further supporting the link between ROS generation and membrane microviscosity alterations. Thus, both the greater generation of ROS and the higher saturated lipid levels likely contribute to the elevated lysosomal membrane microviscosities observed in cancerous cells. The high degree of lipid order displayed in cancerous lysosomal membranes may also be biologically significant, as changes in lipid order can affect cell signaling and alter the function of membrane proteins, such as ion channels and transporters.^56^ Furthermore, highly ordered membranes may limit the passive diffusion of chemotherapeutic drugs into the lysosomes as passive diffusion is largely dependent on membrane viscosity. Crucially, the significant differences in BODIPY-Lys fluorescence lifetimes, corresponding to the large differences in microviscosities between cancerous and noncancerous lysosomes, offer potential for detecting malignant phenotypes or assessing cancer-associated cellular abnormalities.

### 2.4 Imaging the Effects of CADs on Lysosomal Microviscosities

Next, we treated MCF-7, HepG2, and RPE-1 cells for 24 h with half-maximum inhibitory concentrations (IC_50_) of two commonly used CADs, Ser (27.5 μM for MCF-7, 21 μM for HepG2, and 21.5 μM for RPE) and Ast (12.6 μM for MCF-7, 11.3 μM for HepG2, and 9 μM for RPE). The IC_50_ value represents the drug concentration required to induce 50% cell death. The IC_50_ concentrations of Ser and Ast in all investigated cell lines were determined by an MTT assay (Figure S11). Ser and Ast belong to the same class of CADs that inhibit the lysosomal enzyme sphingomyelin phosphodiesterase 1 (SMPD1),^57^ responsible for the breakdown of sphingomyelin into ceramide and phosphorylcholine.^57^ The inhibition of SMPD1 reduces the hydrolysis of sphingomyelin, leading to its accumulation in the lysosomes^58^ and a decreased efflux of cholesterol from the lysosomes.^59^ Both Ser and Ast are hypothesized to induce lysosomal membrane permeabilization, leakage, and distortion through sphingomyelin accumulation and the detergent-like effects of CADs.^60^ Therefore, we expected Ser and Ast to increase lysosomal membrane microviscosities due to their ability to increase the concentrations of cholesterol and sphingomyelin, as both lipids are known for their ability to form viscous and ordered lipid phases.^14,50^

**Figure 6:**
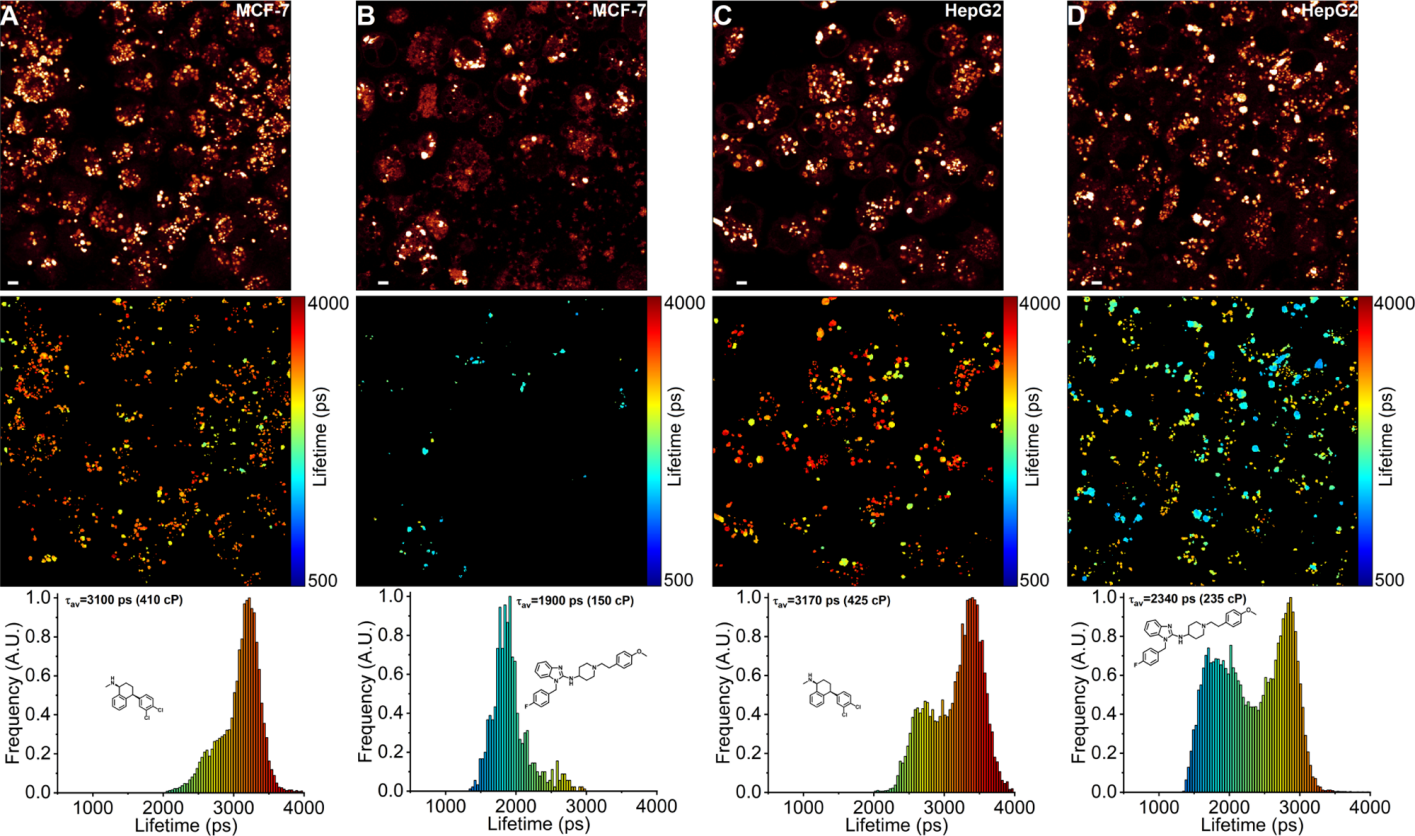
FLIM of BODIPY-Lys in human cancerous cell lines treated with CADs Ser and Ast. (A) MCF-7 cells treated with Ser for 24 h. (B) MCF-7 cells treated with Ast for 24 h. (C) HepG2 cells treated with Ser for 24 h. (D) HepG2 cells treated with Ast for 24 h. The top panel shows images of fluorescence intensity. FLIM images are shown in the middle panel. The corresponding lifetime histograms with CAD structures, mean fluorescence lifetimes τ_av_, and corresponding viscosities in the methanol–glycerol calibration mixtures are shown in the bottom panel. Scale bars are 5 μm.

By performing FLIM of BODIPY-Lys, we observed that Ser-treated MCF-7 and HepG2 cells displayed increased lysosomal microviscosities, with mean intensity-weighted fluorescence lifetimes of BODIPY-Lys increasing from 2400 ps (250 cP) in untreated cells to 3100 ps (410 cP) and 3170 ps (425 cP) in MCF-7 and HepG2 cell lines, respectively (Figure 6A,C). Surprisingly, Ast-treated MCF-7 and HepG2 cells exhibited decreased lysosomal microviscosities, with mean intensity-weighted fluorescence lifetimes of BODIPY-Lys decreasing to about 1900 ps (150 cP) and 2340 ps (235 cP) in MCF-7 and HepG2 cell lines, respectively (Figure 6B,D). Despite both CADs being known for inducing lysosomal membrane permeabilization, leakage, and inhibition of SMPD1,^57^ Ser and Ast exhibited opposite effects on the microviscosities of lysosomal membranes, with only Ast treatment resulting in less viscous, more fluid lysosomal membranes in cancer cells. The microviscosity measurements suggest that the mechanisms by which Ser and Ast permeabilize lysosomal membranes and induce lysosomal leakage may differ. We hypothesize that the different effects of Ast and Ser on the lysosomal membrane microviscosities may arise from the additional detergent-like properties of CADs.^60^ In particular, larger Ast molecules with multiple lipophilic phenyl groups may integrate more easily into the lipid bilayer, exerting stronger detergent effects and creating more disordered, less viscous membranes compared to Ser. Of note, both Ser and Ast treatments resulted in larger lysosomes in cancer cells, likely due to inhibited lipid efflux and subsequent lipid accumulation (Figures 6 and S12). Moreover, in both Ser- and Ast-treated cells, lysosomes exhibited an irregular cellular distribution, formed clusters, and adopted irregular, noncircular shapes. Additionally, particularly large lysosomes in HepG2 cells showed discontinuities in BODIPY-Lys fluorescence intensities, likely due to ruptured lipid bilayers (Figures 6 and S10). We hypothesize that Ser-induced lysosomal leakage occurs through the distortion of lysosomal membranes and the formation of pores in the lipid bilayer. In contrast, Ast may induce lysosomal leakage through both the distortion and pore formation in the lysosomal membranes as well as by directly permeabilizing the lipid bilayer through the formation of an Ld lipid phase. Disordered lipid bilayers are generally more permeable to small solutes due to their increased fluidity, which allows for greater movement of molecules across the membrane.^61^

Interestingly, both Ast and Ser treatments resulted in a heterogeneous population of lysosomes with varying microviscosities in cancer cell lines (Figure 6). In both HepG2 and MCF-7, lysosomes with the highest microviscosities consistently showed reduced BODIPY-Lys accumulation, indicated by decreased fluorescence intensities relative to those of less viscous lysosomes. We attribute this reduced accumulation of BODIPY-Lys in highly viscous lysosomes to an increase in lysosomal pH rather than highly ordered membranes hindering probe penetration. This is supported by the fact that Ast-treated cancer cells, despite not displaying high lysosomal microviscosities, still showed uneven BODIPY-Lys fluorescence intensities, with more viscous lysosomes consistently displaying lower fluorescence signals (Figure 6B,D). The increase in lysosomal pH in CAD-treated cells is a well-known effect, driven in part by the basic nature of CADs and their membrane-permeabilizing effects, which facilitate the escape of H^+^ ions.^62^ Since BODIPY-Lys uptake is pH-dependent, its accumulation decreases in lysosomes with higher pH levels, as the morpholine group of BODIPY-Lys is less likely to become protonated. We hypothesize that the heterogeneous populations of lysosomes with two distinct microviscosities in Ast- and Ser-treated cancer cells appear due to the continuous formation of new lysosomes. As CADs increase the lysosomal pH, it is likely that lysosomes with the lowest fluorescence intensities of BODIPY-Lys have accumulated higher amounts of CADs, while lysosomes with the highest BODIPY-Lys fluorescence intensities are likely newly formed and have not yet experienced a CAD-induced pH increase. In Ser-treated cancer cells, BODIPY-Lys fluorescence lifetimes ranging from 2000 to 3000 ps likely indicate newly formed lysosomes, which share similar fluorescence lifetimes to those in untreated cells (Figure 6A,C). In contrast, lysosomes with BODIPY-Lys fluorescence lifetimes between 3000 and 4000 ps have likely accumulated significant amounts of Ser, leading to an increase in pH, reduced BODIPY-Lys fluorescence intensities, and inhibited cholesterol efflux, which in turn increase the microviscosity of lysosomal membranes. Similarly, in Ast-treated cells, lysosomes with the highest microviscosities show decreased fluorescence intensities, indicating a high accumulation of Ast (Figure 6B,D). However, unlike in Ser-treated cells, Ast treatment leads to a reduction in lysosomal microviscosity in cancer cells. We believe Ast exerts a stronger detergent-like effect on lysosomal membranes compared with Ser, initially reducing their microviscosity. Only after a significant buildup of cholesterol and sphingomyelin occurs, as a result of SMPD1 inhibition, do the fluorescence lifetimes of BODIPY-Lys rise to the 2000–3500 ps range (Figure 6B,D). Thus, Ast likely initially disrupts the lipid bilayers of lysosomal membranes through its detergent action, with the subsequent accumulation of cholesterol and sphingomyelin, leading to increased microviscosities. In contrast, Ser likely does not cause an initial disruption of the lipid bilayer, and the accumulation of cholesterol and sphingomyelin alone results in the observed increase in microviscosities.

Similar to the cancerous cell lines, a 24 h treatment of RPE-1 cells with Ser resulted in increased lysosomal microviscosities (Figure S14), with mean BODIPY-Lys fluorescence lifetimes increasing from 1500 ps (90 cP) to 2620 ps (300 cP). Given that the lysosomes in RPE-1 cells are nonviscous and presumably contain modest levels of saturated and oxidized lipids,^12,13^ the accumulation of sphingomyelin and cholesterol following Ser treatment is likely insufficient to create the almost Lo lysosomal membranes observed in Ser-treated cancer cells. In contrast to the cancer cell lines, Ast treatment of RPE-1 cells resulted in slightly increased lysosomal microviscosities, with mean intensity-weighted fluorescence lifetimes of BODIPY-Lys increasing from 1500 ps (90 cP) to 1700 ps (120 cP). Most likely, the accumulation of sphingomyelin and cholesterol in the initially unsaturated and nonoxidized lysosomal lipid bilayer overcomes the detergent action of Ast, resulting in increased lipid bilayer microviscosities. Additionally, CAD-treated RPE-1 cells did not exhibit significant variations in lysosomal microviscosities (Figure S14), indicating that nearly all lysosomes respond similarly to the particular CAD treatment. Such microviscosity uniformity may be a result of slower lysosomal biogenesis in noncancer cells compared to malignant ones.^4^ Finally, the fluorescence intensities of BODIPY-Lys in CAD-treated RPE-1 cells were uniform, suggesting that the pH values in different lysosomes are comparable (Figure S14).

## 3 Conclusions

In conclusion, we synthesized and investigated the photophysical properties of a new BODIPY-based molecular rotor, BODIPY-Lys. We showed that fluorescence decays of BODIPY-Lys are sensitive to viscosity over a large range, from 0.5 to 1457 cP, while remaining largely unaffected by solvent polarity and temperature. Through time-resolved fluorescence measurements on GUVs with Lo and Ld lipid phases, we demonstrated that BODIPY-Lys exhibits significantly different fluorescence lifetimes in Lo and Ld environments, enabling it to distinguish lipid phase transitions and assess cholesterol-induced changes in the Ld phase properties. Notably, BODIPY-Lys preferentially partitions into the lysosomes of live cells with minimal staining of the cytoplasm or plasma membrane. Our findings reveal that lysosomal membranes in cancerous cells exhibit about three times greater microviscosities compared to those in noncancerous cells, enabling the identification of cancerous cells through the imaging of lysosomal membrane microviscosities. Furthermore, our exploration of the effects of CADs Ser and Ast on cancerous and noncancerous cell lines has revealed that despite their functional similarity, these CAD exert opposite effects on the microviscosities of lysosomal membranes in cancerous cells, with Ser increasing the microviscosities and Ast decreasing them. Additionally, we discovered that in cancerous cells, both CADs induce two distinct populations of lysosomes with differing lipid phases, whereas CAD-treated noncancerous cells display relatively homogeneous lysosomal microviscosities. Our findings not only advance the understanding of lysosomal dynamics but also provide insights that could guide future research into the interplay between lipid composition and cellular health.

## 4 Materials and Methods

### 4.1 Dyes, Reagents, and Solvents

The synthetic details of BODIPY-Lys, with mass and NMR spectra, are presented in the Supporting Information. NMR spectra were recorded on a Bruker Ascend 400 spectrometer (400 MHz for ^1^H, 100 MHz for ^13^C, 128.4 MHz for ^11^B, 376.5 MHz for ^19^F). NMR spectra were referenced to residual solvent peaks. HRMS spectra were recorded on a quadrupole time-of-flight mass spectrometer (microTOF-Q II, Bruker Daltonics). Column chromatography was performed using silica gel 60 (0.040–0.063 mm) (Merck). Thin-layer chromatography (TLC) was performed using TLC-aluminum sheets with silica gel (Merck 60 F254). Visualization was accomplished by using UV light. Melting points were determined in open capillaries with a digital melting point IA9100 series apparatus (Thermo Fisher) and were not corrected. Reagents and solvents for the organic synthesis of the BODIPY compounds were purchased directly from commercial suppliers; solvents were purified using known procedures. Stock solutions of 1 mM BODIPY-Lys were prepared in methanol or DMSO and diluted for further experiments in solvents or their mixtures. All solvents used were of spectroscopic grade and obtained from Sigma-Aldrich. DOPC, DPPC, and cholesterol were obtained from Avanti Polar Lipids. Ser and Ast were obtained from Sigma-Aldrich, and 10 mM stock solutions of Ser and Ast were prepared in DMSO and diluted for subsequent experiments. Neutral Red and DCFH-DA were purchased from Sigma-Aldrich. 10 and 5 mM stock solutions of Neutral Red and DCFH-DA were prepared in DMSO and diluted for use in live cell imaging staining.

### 4.2 Formation of GUVs

GUVs were formed by mixing lipids in appropriate ratios, followed by the addition of BODIPY-Lys at a dye-to-lipid ratio of 1:800. The resulting chloroform solution was then deposited onto a clean indium tin oxide (ITO) slide and evaporated under a nitrogen stream for at least 2 h. After drying, a chamber was assembled by using two ITO slides separated by a spacer and filled with a 400 mM sucrose solution. Electroformation was carried out by applying a sinusoidal voltage of 1 V at 10 Hz for 2 h, followed by a 30 min detachment phase at 2 Hz. For the DOPC/DPPC/Chol (1/5/5) mixture, electroformation was performed at an amplitude of 2.5 V and above the lipid melting temperature.

### 4.3 Absorption, Steady-State, and Time-Resolved Fluorescence

Absorption spectra were measured by using a Jasco V-670 spectrophotometer. Fluorescence spectra were recorded with an Edinburgh-F900 (Edinburgh Instruments) spectrophotometer using a Fianum white laser, together with band-pass filters (Thorlabs), emitting at 488 nm as an excitation source. Fluorescence decays were measured by using time-correlated single-photon counting. Fluorescence decays had 5000 counts at the peak of the decay, with a 20 ns time window and 4096 channels in the time domain. 10 mm quartz cuvettes were used for absorption and fluorescence measurements, with BODIPY-Lys concentrations of up to 2 μM. Fluorescence decays of BODIPY-Lys in solvent mixtures and pure solvents were taken at 20 °C. The instrument response function (IRF) was recorded using a scattering sample, and its full width at half-maximum was 415 ps.

### 4.4 Imaging of Live Cells

RPE-1, HepG2, A549, U-87, HEK 293T, MCF-7, and WPMY-1 cell lines were purchased from ATCC, and HMF cells were purchased from the National Cancer Institute of Lithuania. All of the imaged cells were cultured in Dulbecco’s modified Eagle’s medium supplemented with 10% fetal bovine serum (FBS), 100 IU/mL penicillin, and 100 μg/mL streptomycin (Thermo Fisher). The cells were incubated at 37 °C in 5% CO_2_. Before imaging, cells were seeded into an Ibidi μ-Dish (Ibidi) at a seeding density of 10,000 cells/mL and allowed to grow for 24 h. For cell imaging, a 0.5 μM BODIPY-Lys solution (in DMSO) was added to the culture medium for 5 min at 37 °C. FLIM imaging was done at room temperature using a Leica SP8 microscope with a 63x objective (HC PL APO oil immersion, N.A. −1.4, Leica). For ROS imaging, cells were incubated with 5 μM DCFH-DA in an FBS-free medium for 30 min, followed by replacement with a fresh cell medium.

### 4.5 FLIM

FLIM was done with a Leica SP8 microscope using a 63x objective (HC PL APO oil immersion, N.A. −1.4, Leica). The fluorescence decay signal was measured over the 505–550 nm range by using a 488 nm filter-supported white light laser excitation line. The FLIM images were produced in 512 × 512 pixel resolution with 128 channels in the time domain. The pixels were binned to have at least 1000 counts at the peak of the decay curve for reliable biexponential fitting. Only lysosome-containing pixels in the FLIM images were fitted. Lysosomes were identified based on their 100-fold greater fluorescence counts per pixel relative to the cytoplasm. The IRF was measured by recording the laser reflection signal on a glass coverslip.

### 4.6 Data Analysis

FLIM images were analyzed with FLIMFIT software (v4.6.1, Imperial College London). The biexponential fluorescence decay model with intensity-weighted mean lifetimes (eq 2) was applied for FLIM measurements:
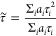
2where *a*_*i*_ and τ_*i*_ are the amplitudes of the individual components. The goodness-of-fit parameter (χ^2^) was 1.3 or less.

## Supporting Information Available

BODIPY-Lys spectra: fluorescence and absorption in various solvents (Figures S1 and S2); influence of protonation (Figure S3); temperature-dependent fluorescence decays in DMSO (Figure S4); NMR and HRMS (Figures S16–S20); microscopy: colocalization of BODIPY-Lys and Neutral Red (Figure S6); bright-field images after BODIPY-Lys addition (Figure S7); assessment of ROS levels (Figure S10); high-magnification images of lysosomal morphology (Figure S12); imaging of CADs in the noncancerous RPE-1 cell line (Figure S14); MTT assays (Figure S11); and synthesis of BODIPY-Lys (Figure S15) (PDF)
