# Supplementary material for: Cancer Cell Identification via Lysosomal Membrane Microviscosities Using a Green-Emitting BODIPY Molecular Rotor
Source: JACS Au. 2025 Apr 14;5(4):2004–14. doi: 10.1021/jacsau.5c00253 (PMC12042019; doi:10.1021/jacsau.5c00253)
Supplement: Supplementary file 1 — au5c00253_si_001.pdf [file au5c00253_si_001.pdf]

Supporting Information for:

Cancer Cell Identification via Lysosomal Membrane Microviscosities Using a Green-Emitting BODIPY Molecular Rotor

Rūta Bagdonaitė, [a] Rokas Žvirblis,[b] Jelena Dodonova-Vaitkūnienė[c] and Artūras Polita*[a,d]

[a.] *Department of Biospectroscopy and bioelectrochemistry, Institute of Biochemistry, Life Sciences Center, Vilnius University, Saulėtekio av. 7, Vilnius, LT-10257, Lithuania. E-mail: arturas.polita@gmc.vu.lt.*
[b.] *Department of Biothermodynamics and drug design, Institute of Biotechnology, Life Sciences Center, Vilnius University, Saulėtekio av. 7, Vilnius, LT-10257, Lithuania*
[c.] *Department of Organic chemistry, Faculty of Chemistry and Geosciences, Institute of Chemistry, Vilnius University, Naugarduko st. 24, Vilnius, LT-03225, Lithuania*
[d.] *Department of Organic Chemistry, Center for Physical Sciences and Technology, Saulėtekio av. 3, Vilnius, LT-10257, Lithuania.*

Table of Contents

**Table S1**	Precise monoexponential fluorescence lifetimes of BODIPY-Lys in cyclohexane, toluene, chloroform, dichloromethane, DMSO, and methanol at room temperature.
**Table S2**	Precise fluorescence lifetimes of BODIPY-Lys in methanol-glycerol mixtures at room temperature.
**Table S3**	Precise fluorescence lifetimes of BODIPY-Lys in DMSO at varying temperatures.
**Figure S1**	Absorption, steady-state and time-resolved fluorescence spectra of BODIPY-Lys in various solvents.
**Figure S2**	BODIPY-Lys absorption, steady-state and time-resolved fluorescence spectra in methanol-glycerol mixtures.
**Figure S3**	Influence of morpholine protonation on the photophysical properties of BODIPY-Lys.
**Figure S4**	Time-resolved fluorescence decays of BODIPY-Lys in DMSO at varying temperatures.
**Figure S5**	Time-resolved fluorescence decays of BODIPY-Lys in GUVs.
**Figure S6**	Co-localization of BODIPY-Lys and Neutral Red in HepG2 cells.
**Figure S7**	Brightfield microscopy of various cell lines stained with BODIPY-Lys.
**Figure S8**	Steady-state and time-resolved fluorescence of BODIPY-Lys in lysosomes.
**Figure S9**	Time-resolved fluorescence decays of BODIPY-Lys in lysosomes of HepG2 cells.
**Figure S10**	Assessement of ROS levels in HepG2, U-87, HMF, and WPMY-1 live cells.
**Figure S11**	MTT assay of sertraline (Ser) and astemizole (Ast).
**Figure S12**	High-magnification fluorescence intensity images of BODIPY-Lys in MCF-7 and HepG2 cell lines.
**Figure S13**	Distribution of areas occupied by lysosomes in MCF-7 and HepG2 cells.
**Figure S14**	Imaging the effects of CADs on lysosomal microviscosities of non-cancerous human cell lines (RPE-1).
**Figure S15**	Synthesis of BODIPY-Lys.
**Figure S16**	^{1}H NMR spectrum of BODIPY-Lys.
**Figure S17**	^{13}C NMR spectrum of BODIPY-Lys.
**Figure S18**	^{19}F NMR spectrum of BODIPY-Lys.
**Figure S19**	^{11}B NMR spectrum of BODIPY-Lys.
**Figure S20**	Mass spectrum of BODIPY-Lys.

Absorption, steady-state and time-resolved fluorescence spectra of BODIPY-Lys in methanol, DMSO, dichloromethane, chloroform, toluene, and cyclohexane.

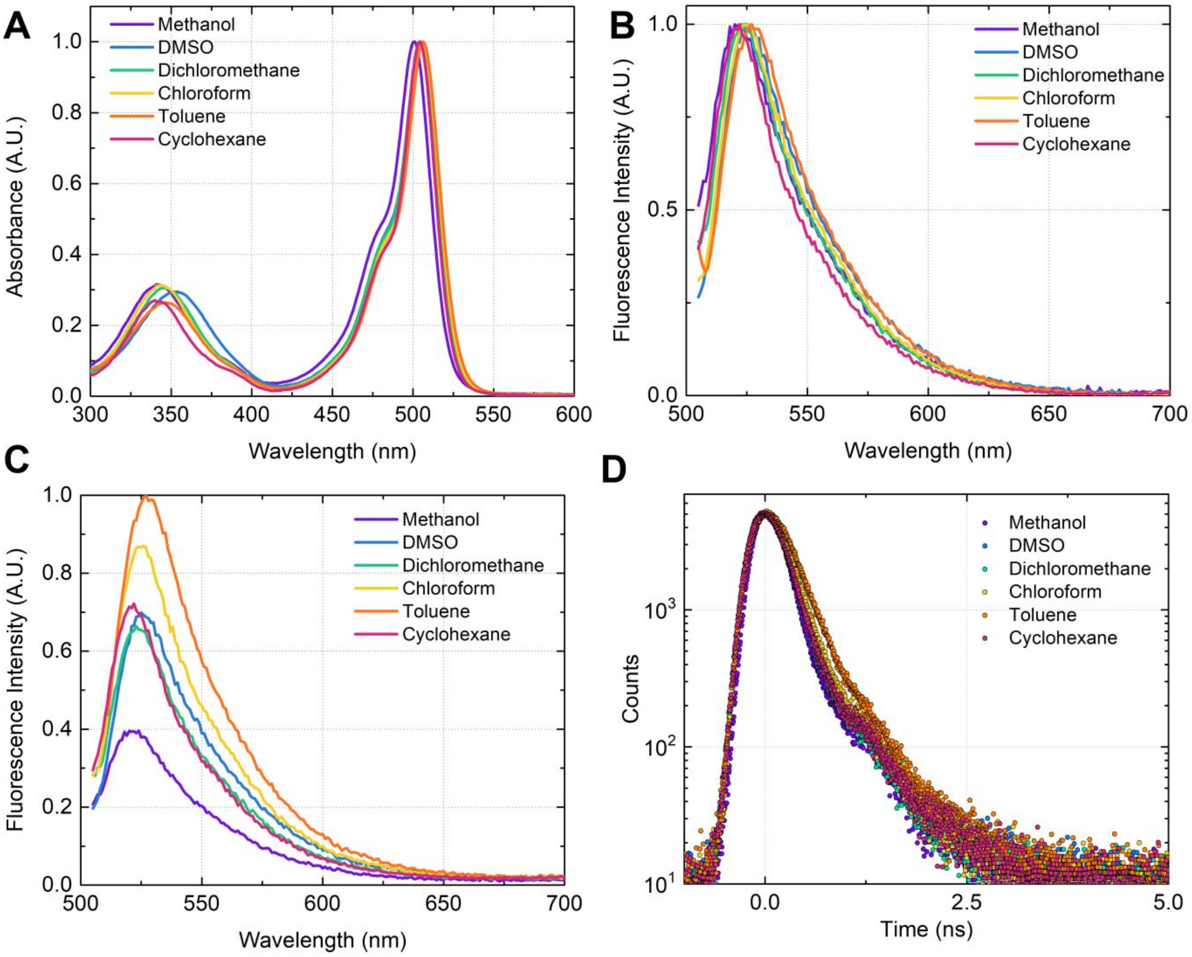

Figure S1. (A) Normalized absorption spectra of BODIPY-Lys in methanol, DMSO, dichloromethane, chloroform, toluene, and cyclohexane. (B) Normalized steady-state fluorescence spectra of BODIPY-Lys in methanol, DMSO, dichloromethane, chloroform, toluene, and cyclohexane. (C) Relative steady-state fluorescence spectra of BODIPY-Lys in methanol, DMSO, dichloromethane, chloroform, toluene, and cyclohexane. (D) Time-resolved fluorescence decays of BODIPY-Lys in methanol, DMSO, dichloromethane, chloroform, toluene, and cyclohexane.

BODIPY-Lys was dissolved at a concentration of 2 μM in various solvents with different polarities (Fig. S1). No visible aggregation of the dye was observed in any of the tested solvents. Additionally, the absence of red-shifted bands in the steady-state emission spectra confirms that BODIPY-Lys does not form aggregates in either low- or high-polarity solvents (Fig. S1B and S1C). The time-resolved fluorescence decays of BODIPY-Lys in cyclohexane, toluene, chloroform, dichloromethane, DMSO, and methanol were monoexponential (Fig. S1D). The precise fluorescence lifetimes are listed in Table S1.

**Solvent**	**Fluorescence lifetime τ, ps**	**χ2**
Methanol	71.18	1.297
DMSO	138.08	1.265
Dichloromethane	115.96	1.267
Chloroform	151.18	1.299
Toluene	203.1	1.292
Cyclohexane	126.81	1.245

Table S1. Precise monoexponential fluorescence lifetimes of BODIPY-Lys in cyclohexane, toluene, chloroform, dichloromethane, DMSO and methanol at room temperature.

BODIPY-Lys absorption, steady-state, and time-resolved fluorescence spectra in methanol-glycerol mixtures.

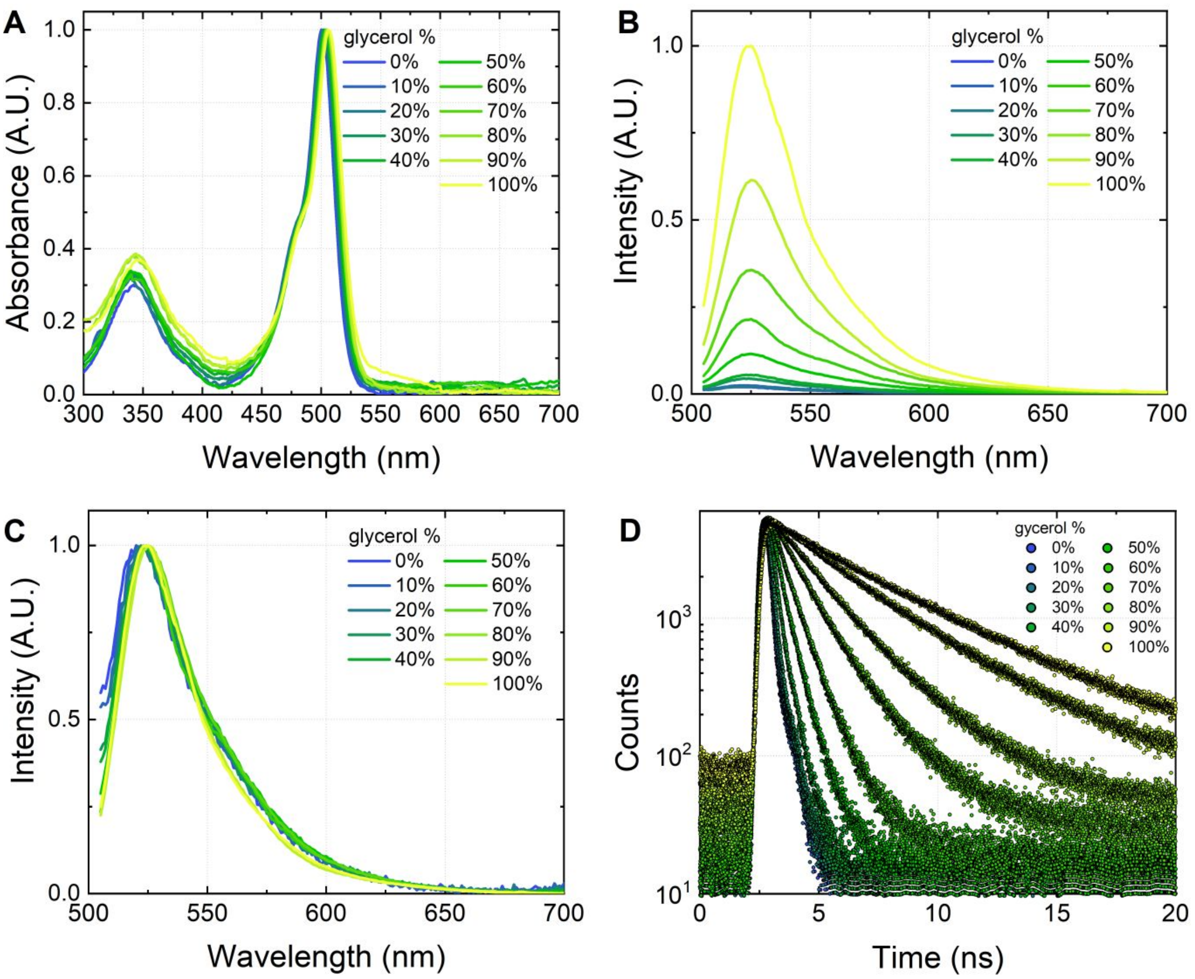

Figure S2. (A) Normalized absorption spectra of BODIPY-Lys in methanol-glycerol mixtures. (B) Relative steady-state fluorescence spectra of BODIPY-Lys in methanol-glycerol mixtures. (C) Normalized steady-state fluorescence spectra of BODIPY-Lys in methanol-glycerol mixtures. (D) Time-resolved fluorescence decays of BODIPY-Lys in methanol-glycerol mixtures.

BODIPY-Lys was dissolved at a concentration of 2 µM in methanol-glycerol mixtures, beginning with pure methanol and progressing to pure glycerol (Fig. S2). The absorption spectra of BODIPY-Lys exhibited a slight redshift with increasing glycerol concentration, shifting from a peak maximum at 500 nm in methanol to 506 nm in pure glycerol (Fig. S2A). No visible aggregation of the dye was observed in any of the mixtures tested. The time-resolved fluorescence decays of BODIPY-Lys were monoexponential in methanol and methanol-glycerol mixtures up to 40% glycerol. However, starting from the 50% methanol-50% glycerol mixture, the fluorescence decays became biexponential (Fig. S2D). Detailed fluorescence lifetimes of BODIPY-Lys and viscosities of the methanol-glycerol mixtures are provided in Table S2.

Composition	τ_1, ps	τ_2, ps	A_1, %	A_2, %	Intensity-weighted fluorescence lifetime, ps	Viscosity, cP	χ2
Methanol	71.18		100			0.627	1.297
MeOH-10% Gly	88.85		100			1.1	1.281
MeOH-20% Gly	109.32		100			2.05	1.271
MeOH-30% Gly	148.00		100			3.33	1.231
MeOH-40% Gly	249.39		100			6.46	1.482
MeOH-50% Gly	365.66	889.73	83.43	16.57	452.51	14.02	1.107
MeOH-60% Gly	509.37	1062.07	59.02	40.98	735.68	30.73	1.078
MeOH-70% Gly	832.18	1844.75	44.48	55.52	1394.38	72.96	1.091
MeOH-80% Gly	1079.84	2492.77	26.58	73.42	2117.19	183.43	1.067
MeOH-90% Gly	1045.04	3749.93	8.12	91.88	3530.29	513.36	1.097
Glycerol	839.18	4700.96	4.04	95.96	4545.03	1457.55	1.091

Table S2. Precise fluorescence lifetimes of BODIPY-Lys in methanol-glycerol mixtures at room temperature.

Influence of morpholine protonation on the photophysical properties of BODIPY-Lys.

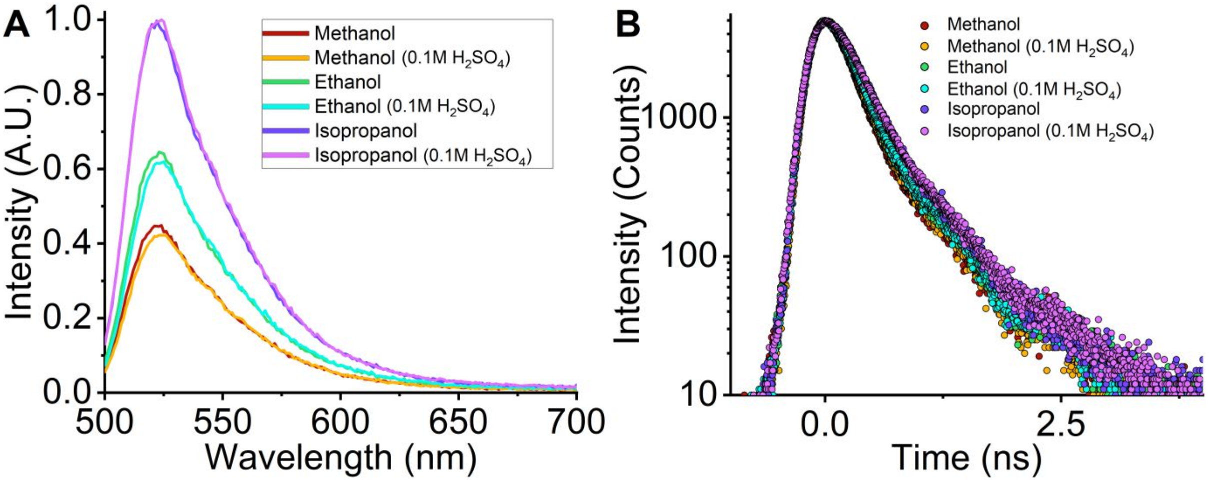

Figure S3. (A) Relative steady-state fluorescence spectra of BODIPY-Lys in methanol, ethanol, and isopropanol, in the presence and absence of 0.1 M sulfuric acid. (B) Time-resolved fluorescence decays of BODIPY-Lys in the same solvents, illustrating the minimal effect of morpholine protonation on BODIPY-Lys fluorescence lifetimes.

To assess the influence of morpholine protonation, we performed steady-state and time-resolved fluorescence measurements in methanol, ethanol, and isopropanol, both with and without 0.1 M sulfuric acid. BODIPY-Lys was dissolved at a concentration of 1 µM in each solvent. The steady-state fluorescence intensities (Fig. S3A) remained unchanged in the presence of sulfuric acid, indicating no significant effect on fluorescence emission. Time-resolved fluorescence measurements showed only minor shifts in BODIPY-Lys fluorescence lifetimes following sulfuric acid addition, increasing from 72 to 79 ps in methanol and from 165 to 169 ps in isopropanol, while slightly decreasing from 117 to 115 ps in ethanol (Fig. S3B). Notably, all fluorescence decays remained monoexponential.

Time-resolved fluorescence decays of BODIPY-Lys in DMSO at varying temperatures.

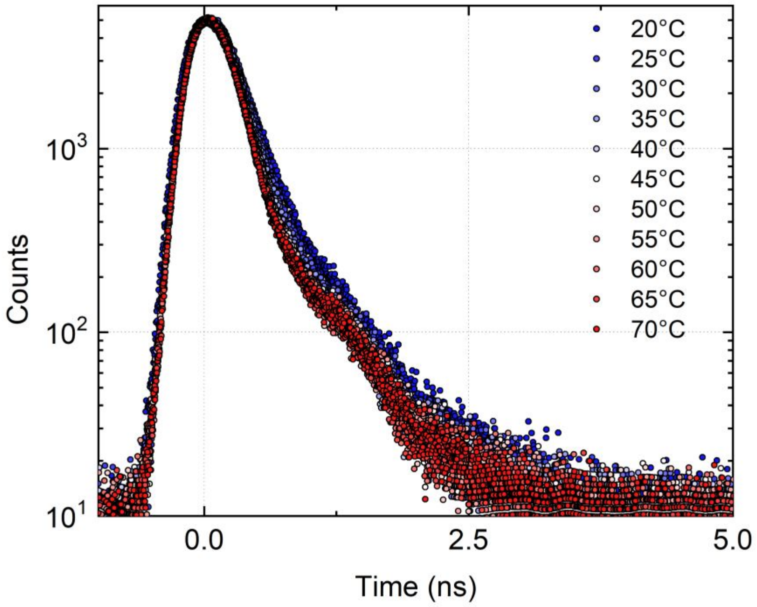

Figure S4. Time-resolved fluorescence decays of BODIPY-Lys in DMSO at varying temperatures (20–70 °C).

BODIPY-Lys was dissolved at a concentration of 2 µM in DMSO, and temperature-dependent time-resolved fluorescence decays were recorded using a water-heated sample holder (Fig. S4). All observed fluorescence decays of BODIPY-Lys were monoexponential. The precise fluorescence lifetimes are provided in Table S3.

**Temperature, °C**	**Fluorescence lifetime τ, ps**	**χ2**
20	187.2	1.443
25.2	136.94	1.309
30.1	129.25	1.29
35.1	119.92	1.325
39.95	112.68	1.255
45.1	108.77	1.354
50.15	103.37	1.311
55	96.35	1.325
60.15	92.8	1.334
65	88.72	1.33
70.3	86.69	1.338

Table S3. Precise fluorescence lifetimes of BODIPY-Lys in DMSO at varying temperatures.

Time-resolved fluorescence decays of BODIPY-Lys in GUVs.

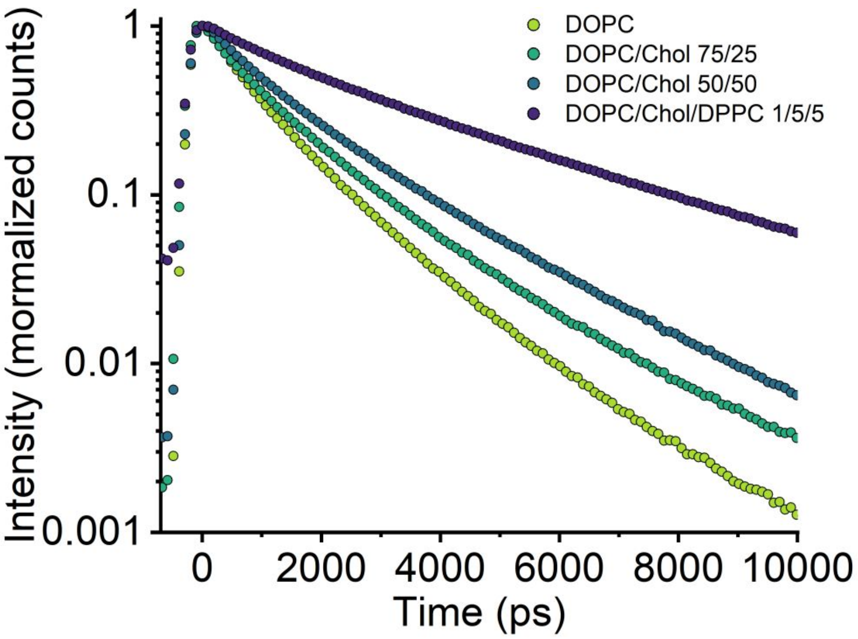

Figure S5. Time-resolved fluorescence decays of BODIPY-Lys in GUVs composed of DOPC, DOPC/Chol (75/25), DOPC/Chol (50/50), and DOPC/Chol/DPPC (1/5/5).

Time-resolved fluorescence decays of BODIPY-Lys in GUVs, selected from Figure 3. The fluorescence decays of BODIPY-Lys in GUVs were biexponential (Fig. S5). The biexponential decay likely reflects the presence of multiple probe positions within the lipid bilayers.

Co-localization of BODIPY-Lys and Neutral Red in HepG2 cells.

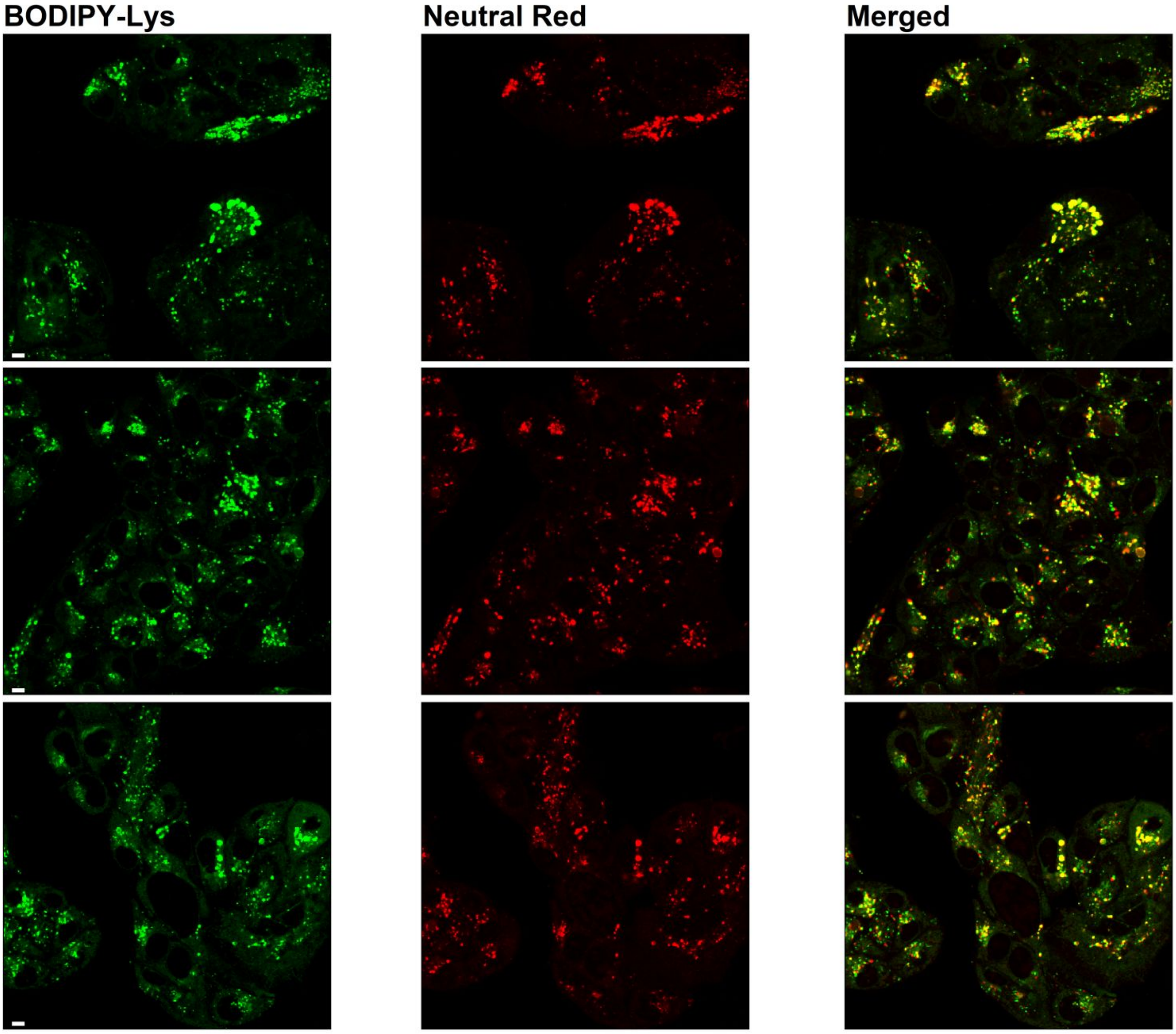

Figure S6. Fluorescence intensity images of HepG2 cells stained with BODIPY-Lys and Neutral Red. Scale bars are 5 μm.

BODIPY-Lys was excited using a 488 nm filter-supported laser line, and fluorescence intensity images were captured in the 505-550 nm range. For Neutral Red, excitation was performed with a 553 nm filter-supported laser line, and fluorescence intensity was recorded in the 650-750 nm range.

Brightfield microscopy of various cell lines stained with BODIPY-Lys.

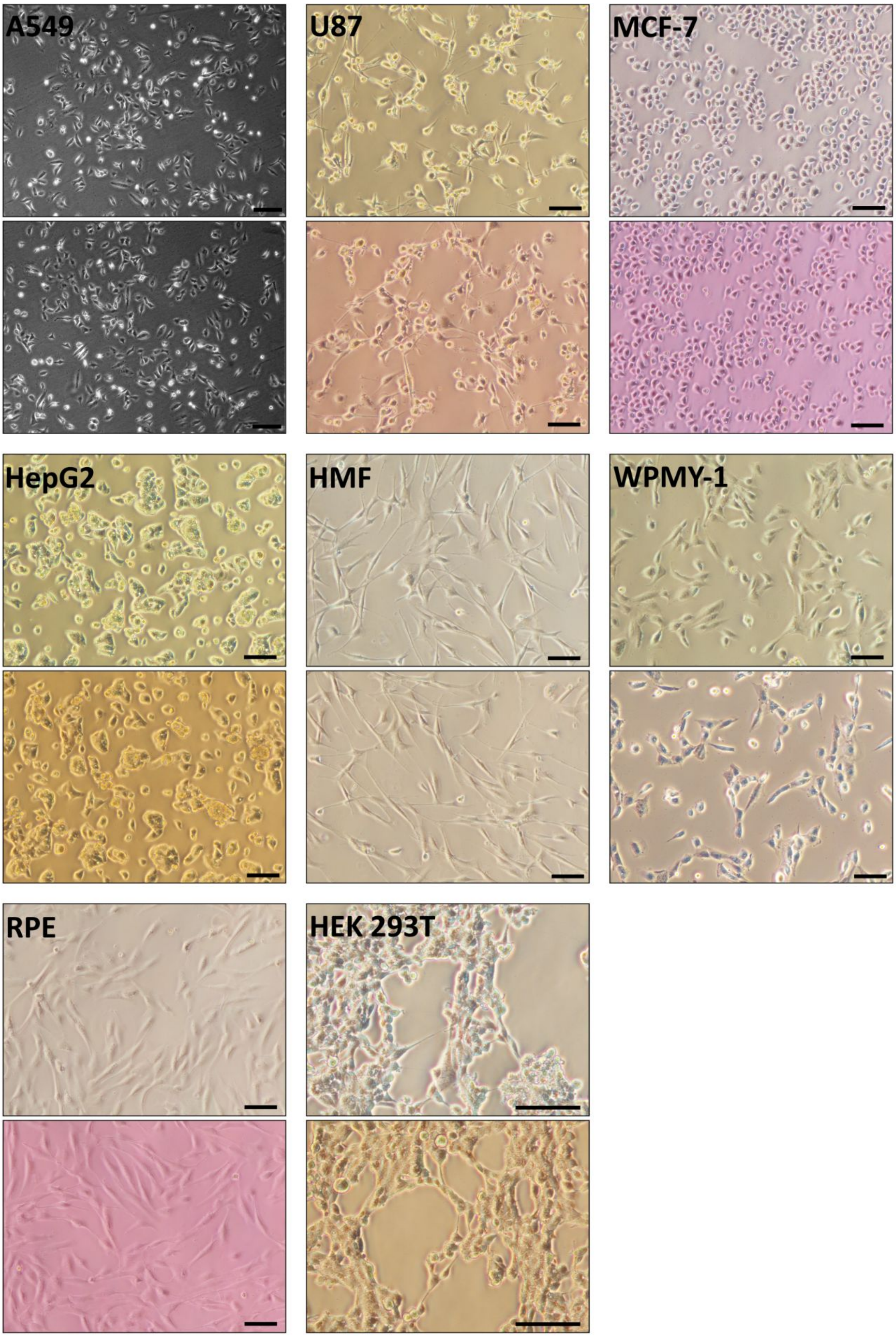

Figure S7. Brightfield images of different cell lines before (upper panel) and 30 min after (bottom panel) addition of BODIPY-Lys (0.5 μM). Scale bars are 100 μm.

Brightfield imaging was used to assess the impact of BODIPY-Lys on cell morphology. All cell lines examined showed normal morphology both before and 30 minutes after the addition of BODIPY-Lys at a concentration of 0.5 µM.

Steady-state and time-resolved fluorescence of BODIPY-Lys in lysosomes.

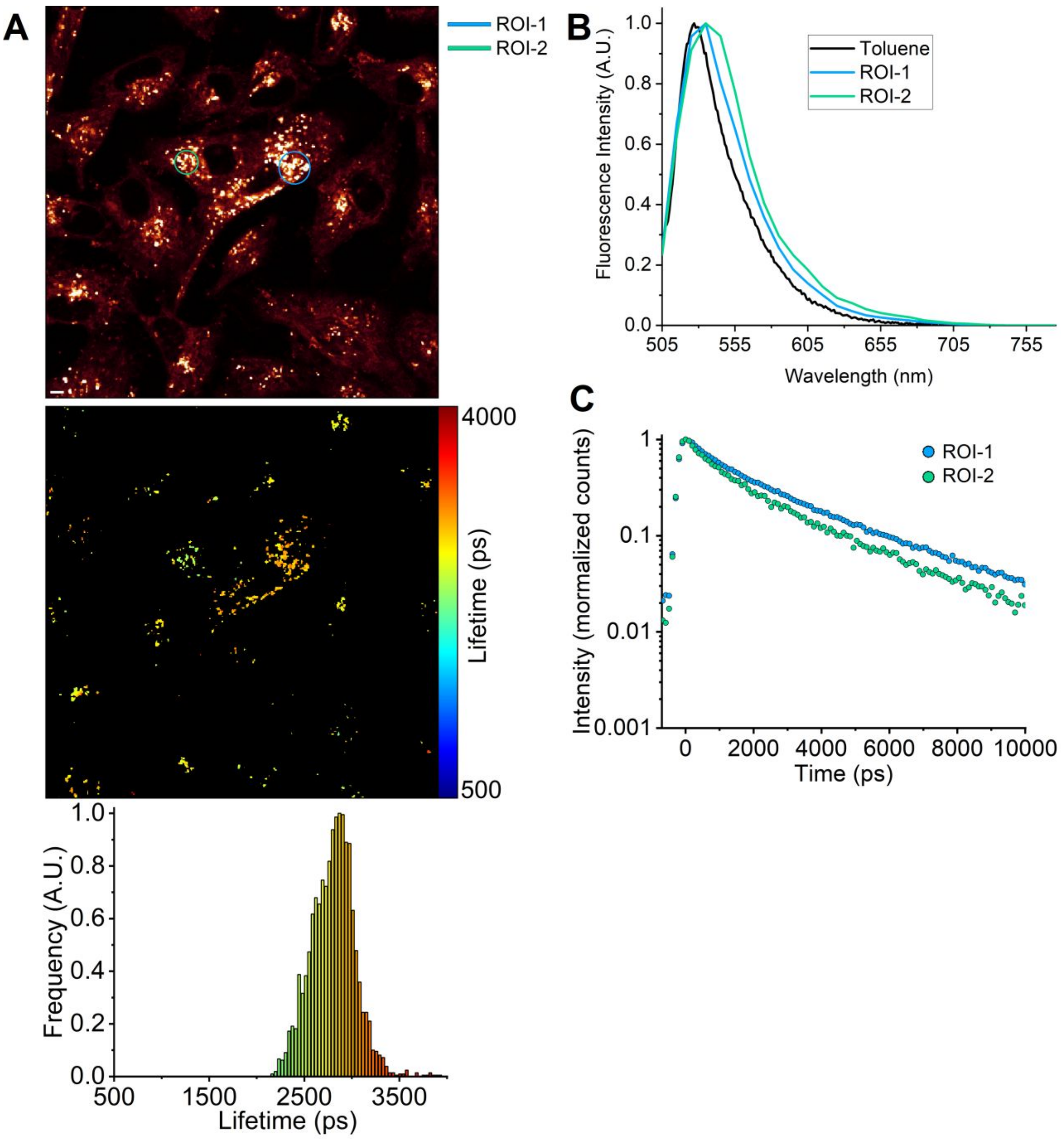

Figure S8. (A) FLIM of BODIPY-Lys (0.5 µM) in A549 cells; the regions of interest (ROIs) from which steady-state fluorescence spectra were recorded are displayed in blue and green in the intensity image. (B) Steady-state fluorescence spectra of BODIPY-Lys in lysosomes and toluene. (C) Time-resolved fluorescence decays of BODIPY-Lys in selected ROIs. Scale bar is 5 µm.

To further confirm the absence of dye aggregation, we recorded steady-state fluorescence spectra of BODIPY-Lys in lysosomes (Fig. S8). The steady-state fluorescence spectra from two distinct regions of interest (ROIs) are presented in Fig. S8B, alongside the toluene reference spectrum. The corresponding ROIs and FLIM data, are displayed in Fig. S8A, while the selected fluorescence decays are presented in Fig. S8C.

Time-resolved fluorescence decays of BODIPY-Lys in lysosomes of HepG2 cells.

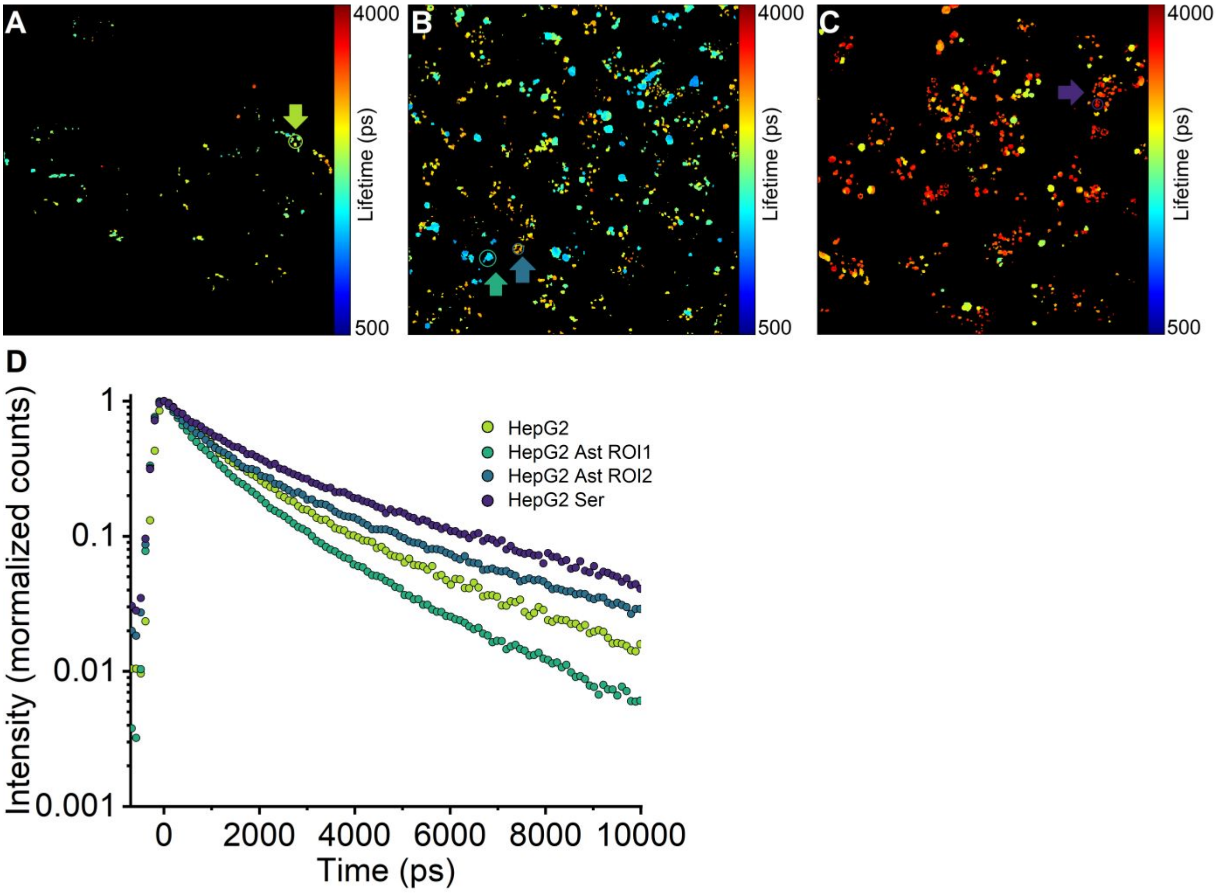

Figure S9. FLIM of BODIPY-Lys (0.5 µM) in HepG2 cells with selected regions of interest (ROIs) indicated by arrows. (A) Untreated HepG2 cells. (B) Astemizole-treated HepG2 cells. (C) Sertraline-treated HepG2 cells. (D) Time-resolved fluorescence decays of BODIPY-Lys in the selected ROIs.

Assesseement of ROS levels in HepG2, U-87, HMF, and WPMY-1 live cells.

To verify the presence of elevated reactive oxygen species (ROS) levels in cancerous cell lines, we performed fluorescence intensity imaging using the commercially available ROS detection probe DCFH-DA. DCFH-DA is a non-fluorescent compound that is deacetylated within cells to form DCFH, which is then oxidized by ROS to produce the green-emitting DCF. HepG2, U-87, HMF, and WPMY-1 cells were stained with 5 µM DCFH-DA for 30 minutes. Fluorescence intensities were imaged using a 488 nm white light laser excitation line, with emission detected in the 510-550 nm window. The laser power and image acquisition time were kept consistent across all measurements to ensure uniform conditions for comparison.

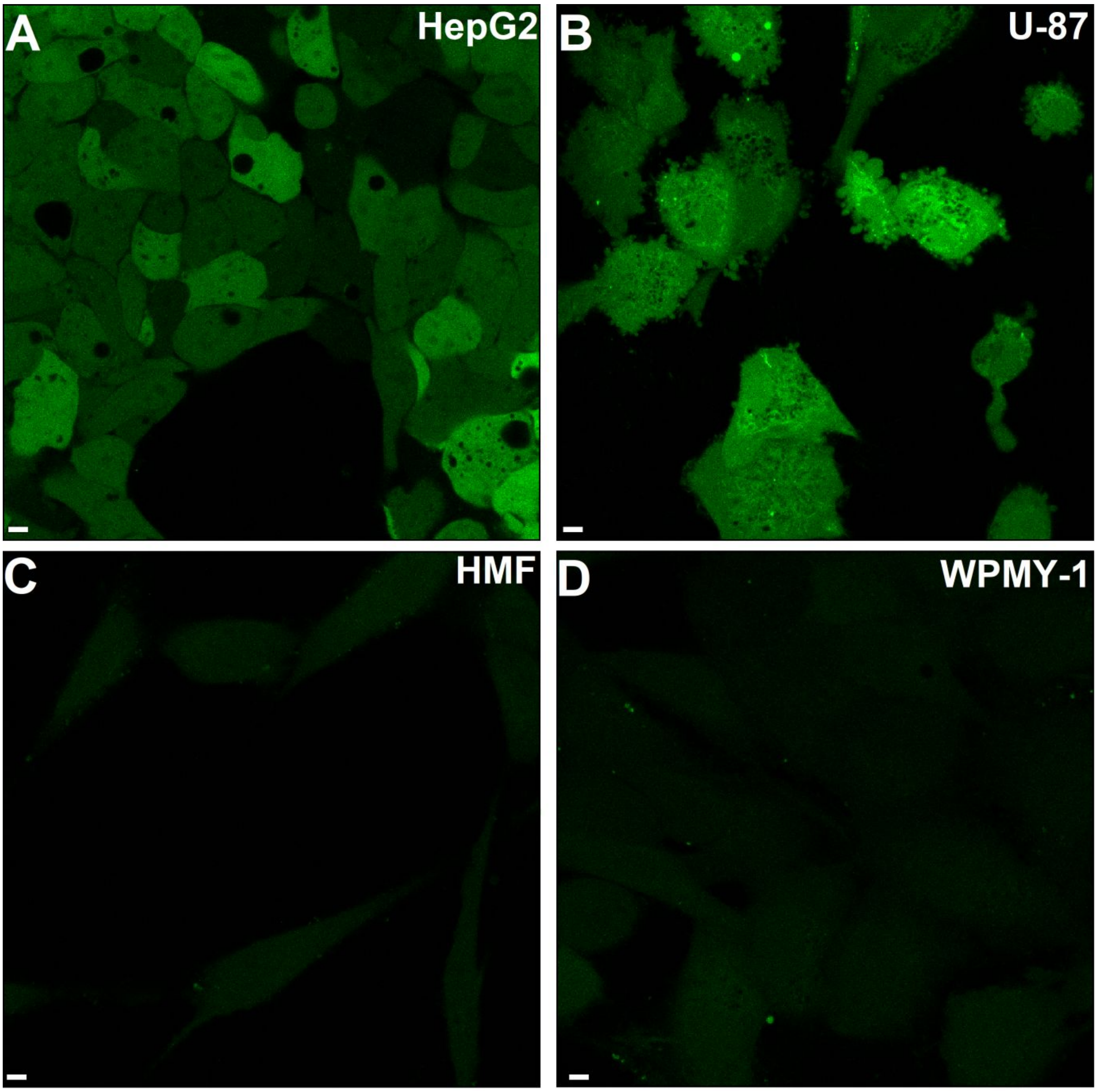

Figure S10. Fluorescence intensity images of DCF in (A) HepG2, (B) U-87, (C) HMF, and (D) WPMY-1 cells. Scale bars represent 5 µm.

The fluorescence intensities of DCF were approximately 5–6 times higher in cancerous HepG2 and U-87 cells compared to non-cancerous HMF and WPMY-1 cells, suggesting elevated ROS generation in malignant cells (Fig. S10). Additionally, DCF fluorescence intensities were uneven in the cancerous cells (Fig. S10A and S10B), indicating variability in ROS levels across different cancer cells.

MTT assay of sertraline (Ser) and astemizole (Ast).

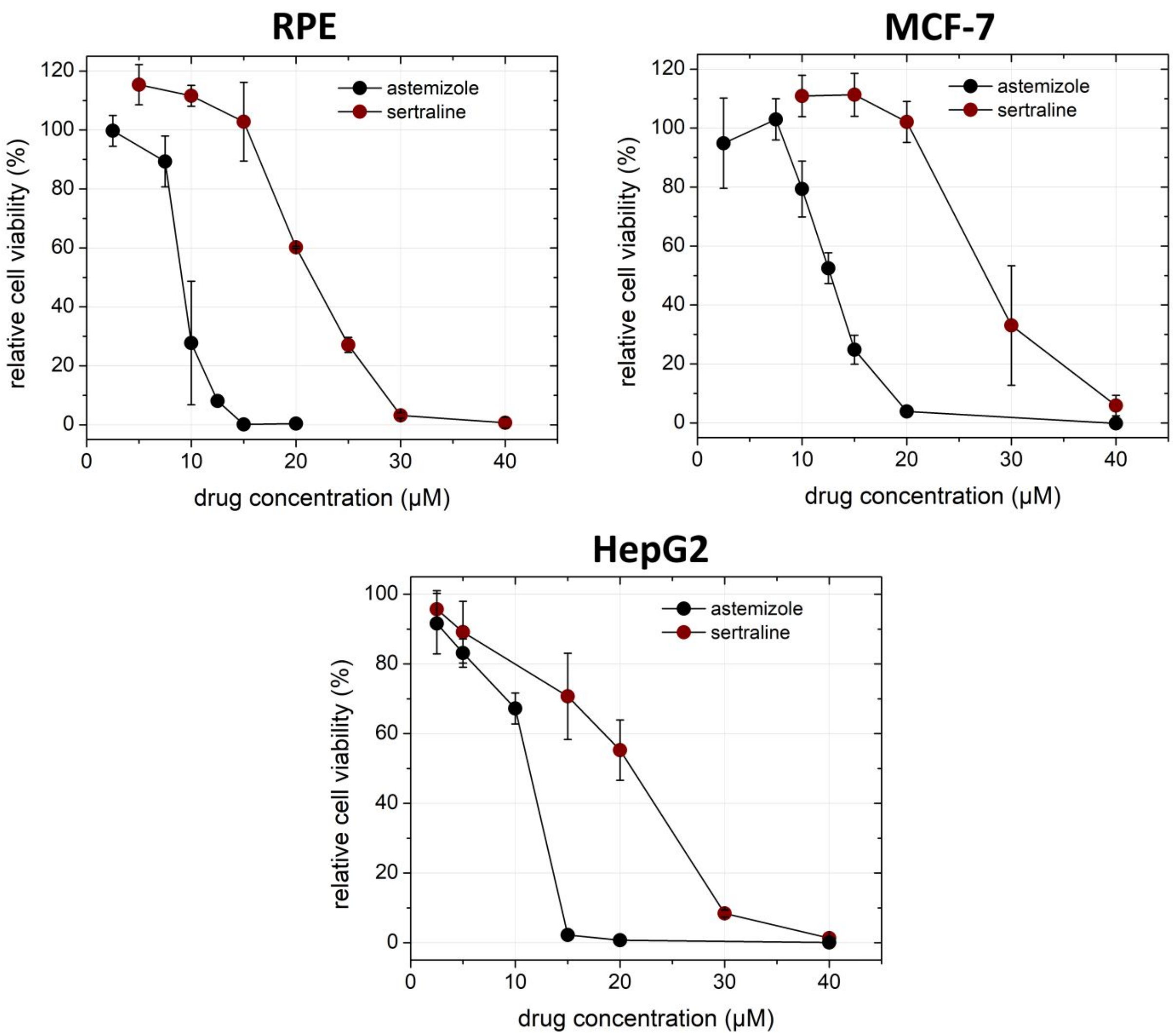

Figure S11. RPE, MCF-7 and HepG2 cell viability after treatment with different concentrations of sertraline and astemizole for 24 h as determined by MTT assay. Results are shown as the mean ± SD of two independent experiments performed in duplicate.

MTT assay was performed to determine the half-maximum inhibitory concentration (IC_{50}) values of sertraline and astemizole on MCF-7, HepG2 and RPE cell lines 24 hours after treatment. The IC_{50} values were 21.5 μM and 9 μM (for RPE), 27.5 μM and 12.6 μM (for MCF-7), 21 μM and 11.3 μM (for HepG2) for sertraline and astemizole, respectively.

Cell culture

All cell lines were cultured in Dulbecco's Modified Eagle Medium (DMEM) supplemented with 10 % fetal bovine serum, 100 units/mL penicillin and 100 μg/mL streptomycin (Thermo Fisher Scientific). Cells were maintained at 37 °C in a humidified 5 % CO_2 incubator. Cells were seeded and allowed to grow for 24 hours in 96-well plates (Thermo Fisher Scientific) for MTT assay and in Ibidi μ-Dish (Ibidi) for imaging.

MTT assay

The half-maximum inhibitory concentration (IC_{50}) values of sertraline and astemizole were determined using MTT assay as described elsewhere [1]. Briefly, 24 hours after drug treatment MTT (Sigma-Aldrich) solution was added to cells at 5 mg/ml and incubated for 1 h. The medium was then removed and the formazan crystals were dissolved in DMSO. Optical density (OD) values at 570 nm were used to calculate cell viability as percentage compared to control cells.

High-magnification fluorescence intensity images of BODIPY-Lys in MCF-7 and HepG2 cell lines.

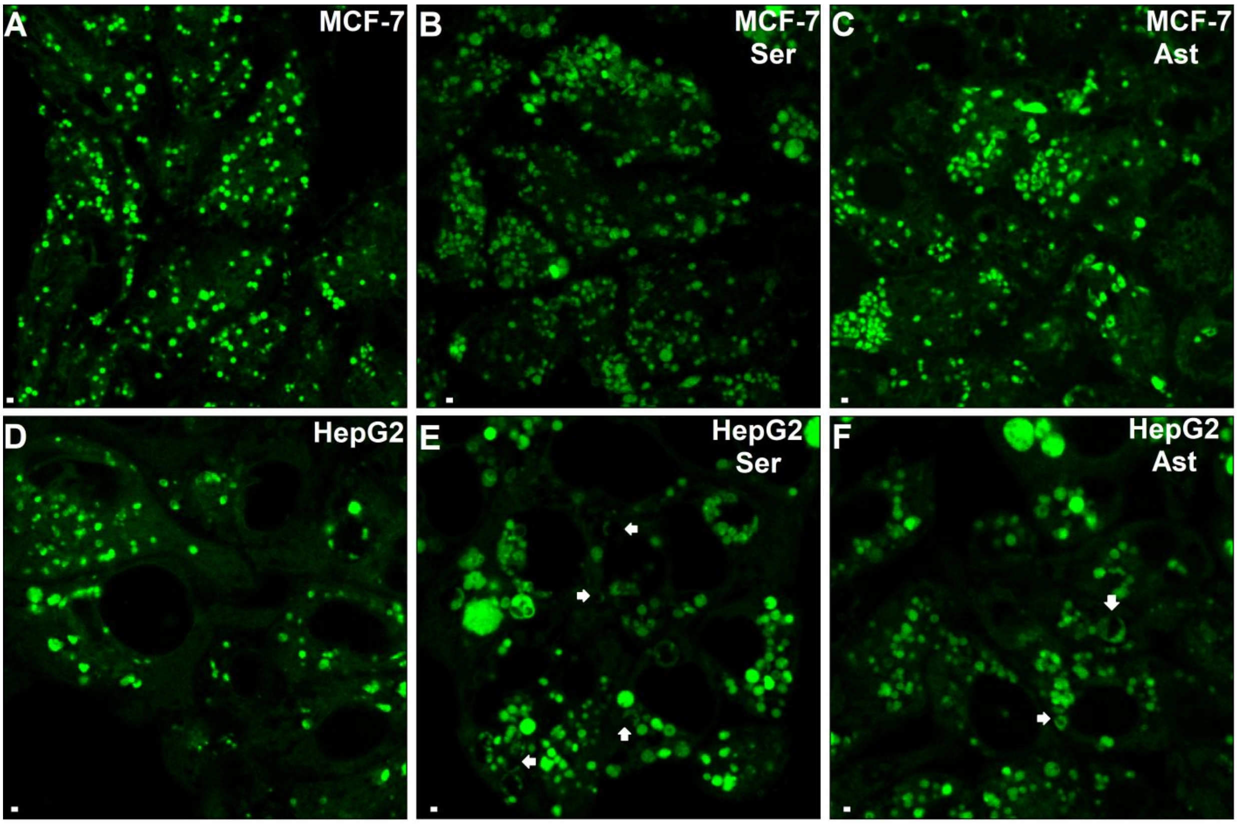

Figure S12. Fluorescence intensity images of BODIPY-Lys in (A) MCF-7 cells, (B) MCF-7 cells treated with sertraline, (C) MCF-7 cells treated with astemizole, (D) HepG2 cells, (E) HepG2 cells treated with sertraline, and (F) HepG2 cells treated with astemizole. Scale bars: 1 µm. Arrows indicate discontinuities in BODIPY-Lys fluorescence intensities.

To assess the morphological changes induced by sertraline and astemizole in lysosomal membranes, we performed high-magnification fluorescence intensity imaging of BODIPY-Lys in MCF-7 and HepG2 cell lines (Fig. S12). Both CADs induced substantial lysosomal clustering, with sertraline and astemizole treatments in HepG2 cells forming large clusters ranging from 3 to 6 µm in size (Fig. S12E and S12F). In contrast, lysosomes in MCF-7 cells clustered near each other but rarely formed large, micron-sized single clusters (Fig. S12B and S12C).

Notably, in HepG2 cells, fluorescence intensity discontinuities in BODIPY-Lys labeling, indicated by arrows, were observed within lysosomal membranes (Fig. S12E and S12F). Such discontinuities may also occur in other cell lines but are likely unresolvable due to optical resolution limitations. Additionally, following CAD treatment, lysosomes in both HepG2 and MCF-7 cells frequently lost their typical circular shape, adopting elongated or irregular morphologies (Fig. S12).

Distribution of areas occupied by lysosomes in MCF-7 and HepG2 cells.

To further evaluate lysosomal size following CAD treatment, we analyzed the intensity images presented in Figure S12, excluding micron-sized lysosomal clusters where individual lysosomes were indistinguishable (Fig. S13). In untreated cells, lysosomal areas averaged 0.38 μm^2 in MCF-7 and 0.57 μm^2 in HepG2 cells, respectively. Sertraline treatment increased lysosomal sizes to 0.54 μm^2 in MCF-7 and 0.94 μm^2 in HepG2, while astemizole treatment resulted in lysosomal sizes of approximately 0.52 μm^2 and 0.80 μm^2 in MCF-7 and HepG2, respectively.

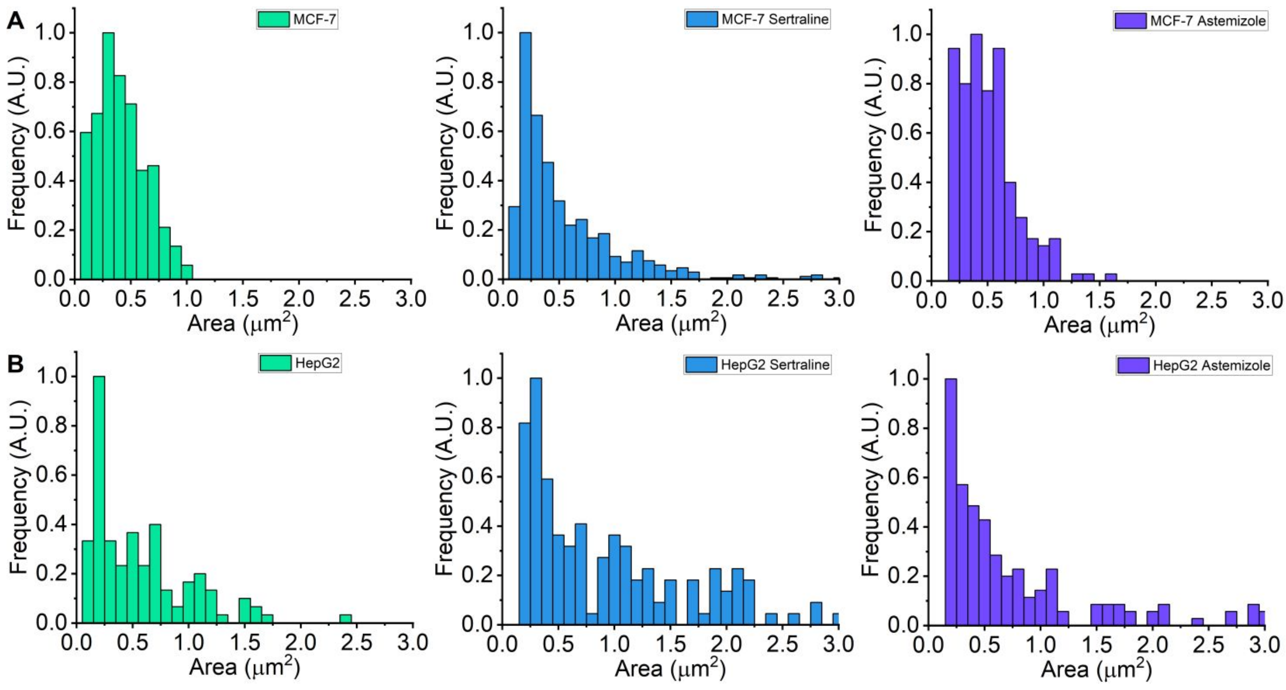

Figure S13. Distribution of areas occupied by lysosomes in (A) MCF-7 and (B) HepG2 cells following treatment with sertraline and astemizole.

Imaging the effects of CADs on lysosomal microviscosities of non-cancerous human cell lines (RPE-1).

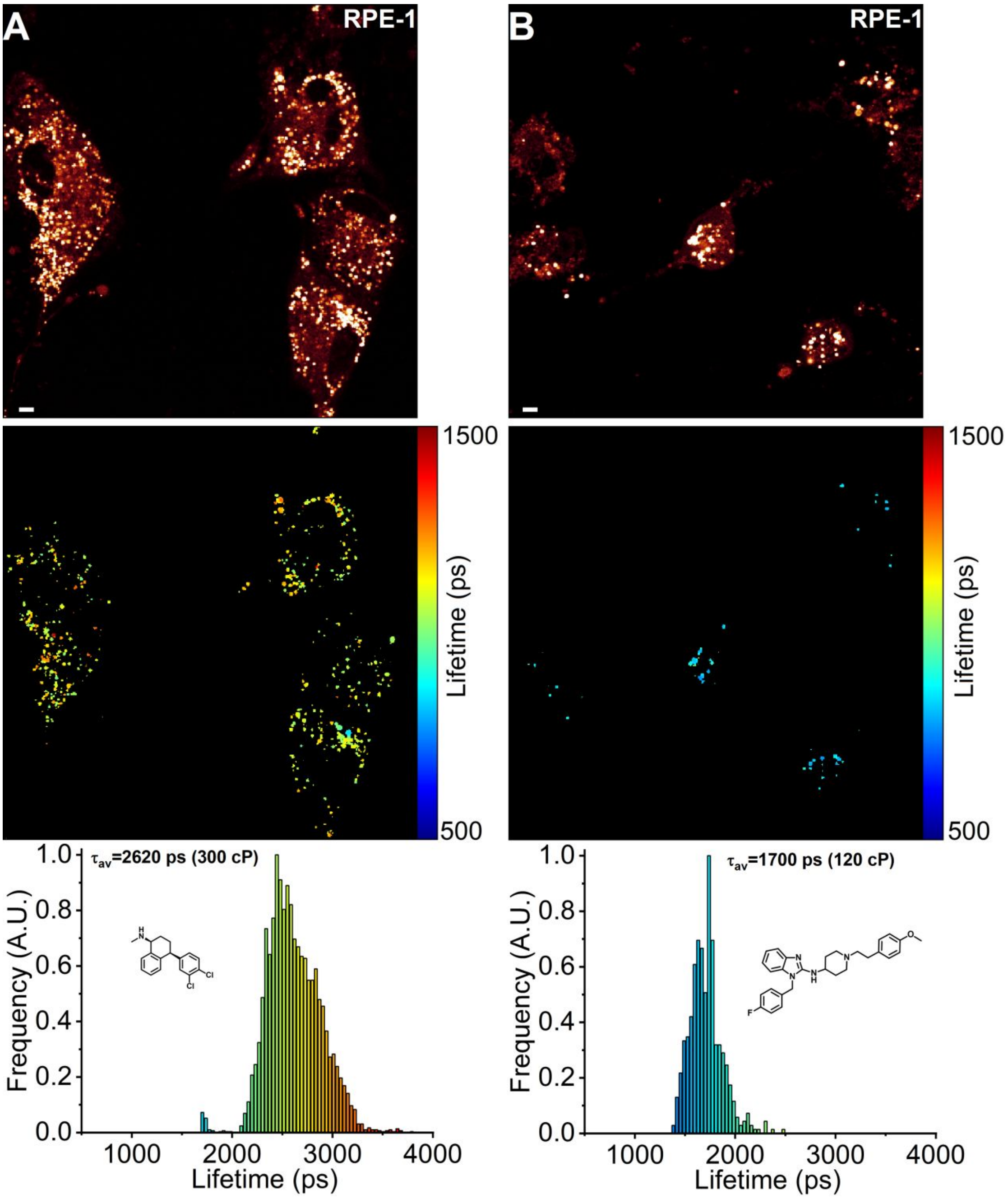

Figure S14. FLIM of BODIPY-Lys in human non-cancerous cell line RPE-1 treated with CADs Ser (A) and Ast (B) for 24 hours. The top panel shows images of fluorescence intensity. FLIM images are shown in the middle panel. The corresponding lifetime histograms with CAD structures, mean fluorescence lifetimes τ_{av} and corresponding viscosities are shown in the bottom panel. Scale bars are 5 µm.

Synthesis of BODIPY-Lys and spectral identification.

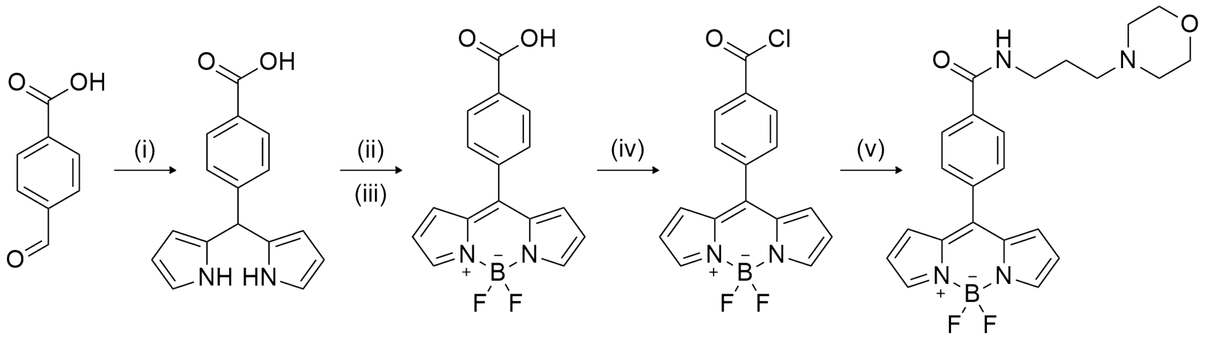

Figure S15. ***Reagents and conditions***: (i) 10 eq. of neat pyrrole, 0.1 M HCl, CH_2Cl_2, argon, r.t., 24 h; (ii) 1.5 eq. DDQ, CH_2Cl_2, and then (iii) 7 eq. $BF_3{\cdot}(OEt_2)_2$ and 7 eq. Et_3N, CH_2Cl_2, argon, 0 ^{o} → r.t., darkness, 24 h; (iv) 2 eq. oxalyl chloride, 4 eq. K_2CO_3, drop of DMF, CH_2Cl_2, argon, r.t., 45 min; (v) 1.33 eq. 3-morpholinopropan-1-amine, 1.33 eq. DIPEA, CH_2Cl_2, argon, r.t., 24 h.

Compounds **A**, **B** [2] and **C** [3] were synthesized according to previously published procedures.

BODIPY-Lys. Compound **C** (50 mg, 0.151 mmol) and 1.33 eq. of 3-morpholinopropan-1-amine (29.51 µl, 0.202 mmol) were dissolved in 2 mL of CH_2Cl_2 and 1.33 eq. of DIPEA (35.2 µL, 0.202 mmol) was added. The mixture was degassed with argon and stirred for 24 hours at room temperature. The reaction progress was controlled using thin-layer chromatography. After the reaction was complete, the solvent was removed under reduced pressure and the crude product was purified by column chromatography on silica gel (eluent – $CHCl_3$→$CHCl_3$:MeOH (9:1)).

BODIPY-Lys. Sticky orange glass, yield 60 mg (91%), mp 66-67 ^oC, ^{1}**H NMR** (400 MHz, $CDCl_3$): δ (ppm) 8.20 (t, *J* = 6 Hz, NH); 8.01-7.98 (m, 4H); 7.63 (d, *J* = 8 Hz, 2H); 6.87 (d, *J* = 4 Hz, 2H); 6.55 (d, *J* = 4 Hz, 2H); 3.71 (t, *J* = 4.6 Hz, 4H); 3.60 (k, *J* = 5.8 Hz, 2H); 2.63-2.51 (m, 6H); 1.84 (p, *J* = 6.2 Hz, 2H). ^{13}**C NMR** (100 MHz, $CDCl_3$): δ (ppm) = 145.9; 144.6; 136.97; 136.39; 134.7; 131.4; 130.6; 127.1; 118.9; 108.0; 66.8; 58.3; 53.7; 40.4; 24.3. ^{11}**B NMR** (128.4 MHz, $CDCl_3$): δ (ppm) = 0.23 (t, $J_{B\text{-}F}$ = 28.9 Hz). ^{19}**F NMR** (376.5 MHz, $CDCl_3$): δ (ppm) = -144.87 (dd, $J_{F\text{-}F}$ = 56.5 Hz, $J_{F\text{-}B}$ = 30.12 Hz).
HRMS (MALDI-TOF) m/z 439.21 ($C_{23}H_{26}BF_2N_4O_2^+$ $[M+H]^+$, requires 439.21).

NMR spectra.

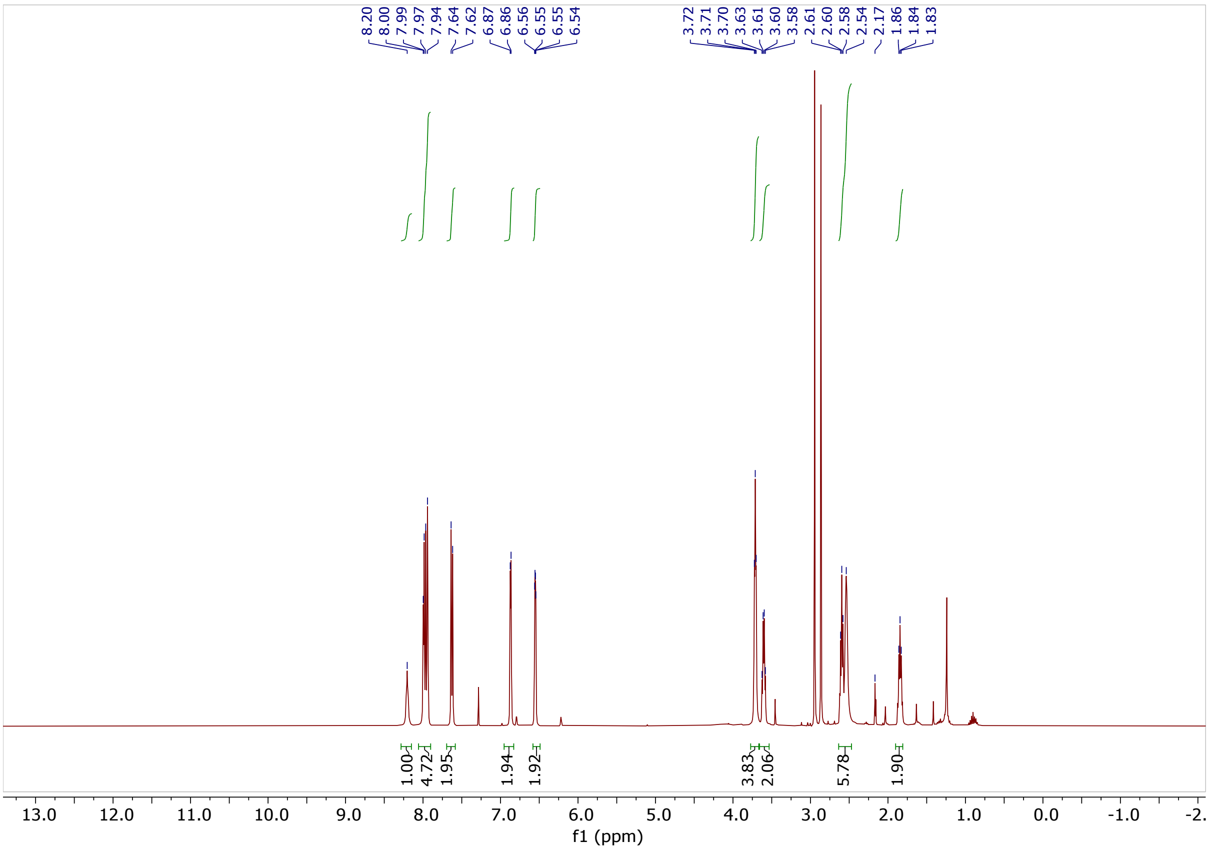

Fig S16. ^{1}H NMR spectrum of **BODIPY-Lys.**

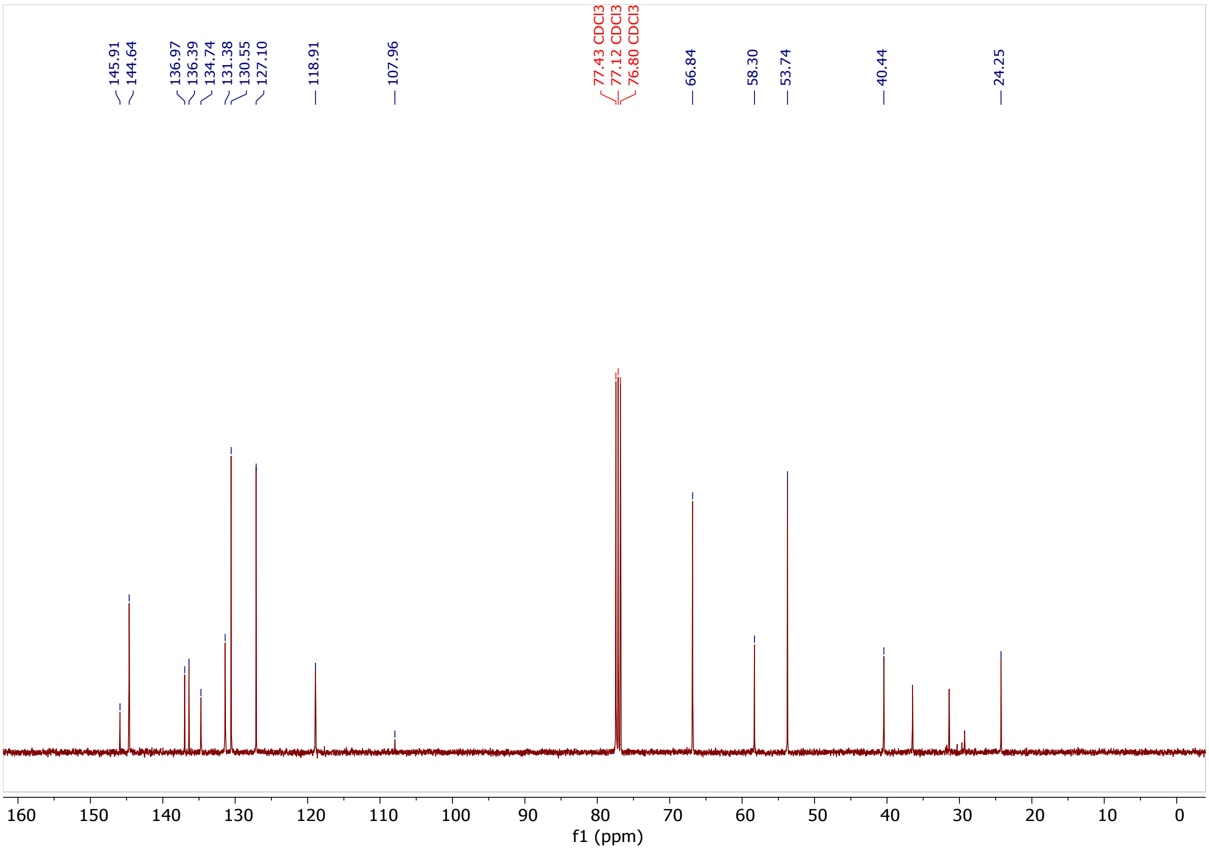

Fig S27. ^{13}C NMR spectrum of **BODIPY-Lys.**

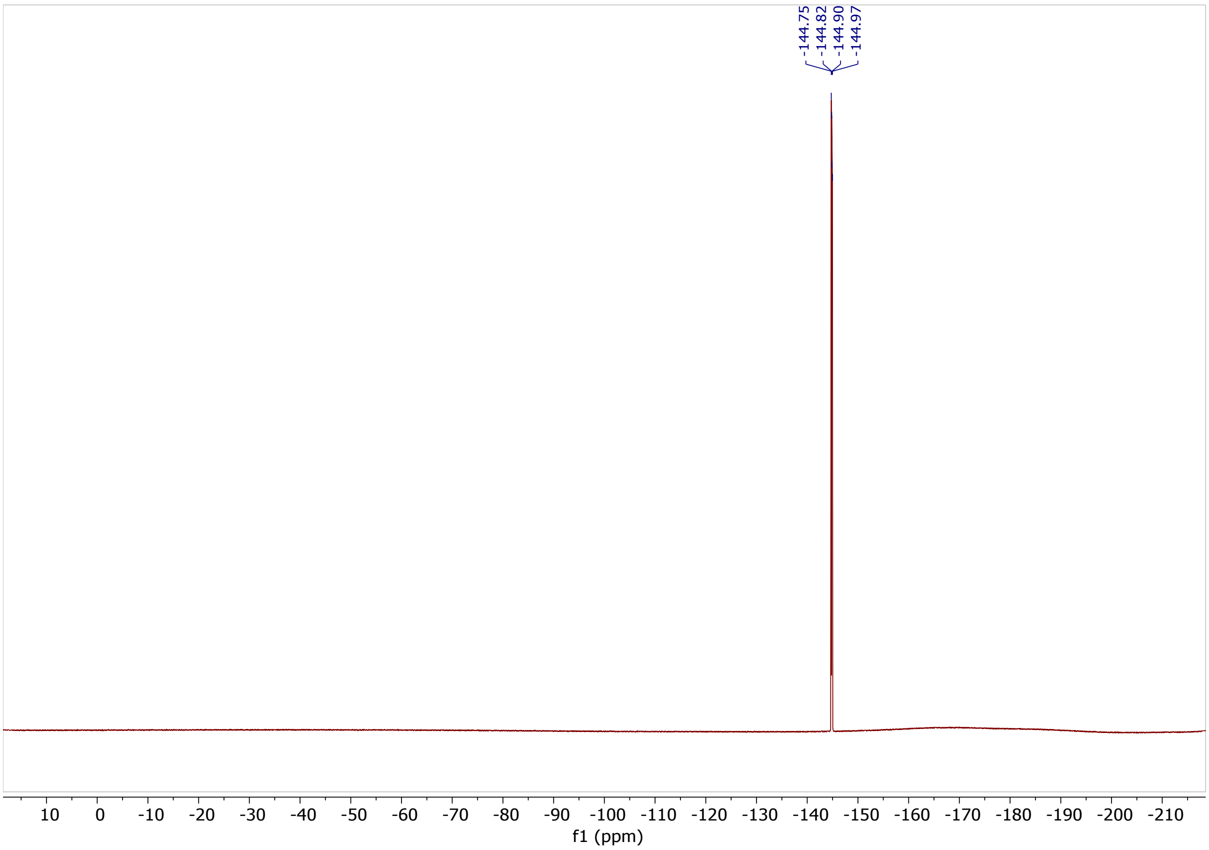

Fig S38. ^{19}F NMR spectrum of **BODIPY-Lys.**

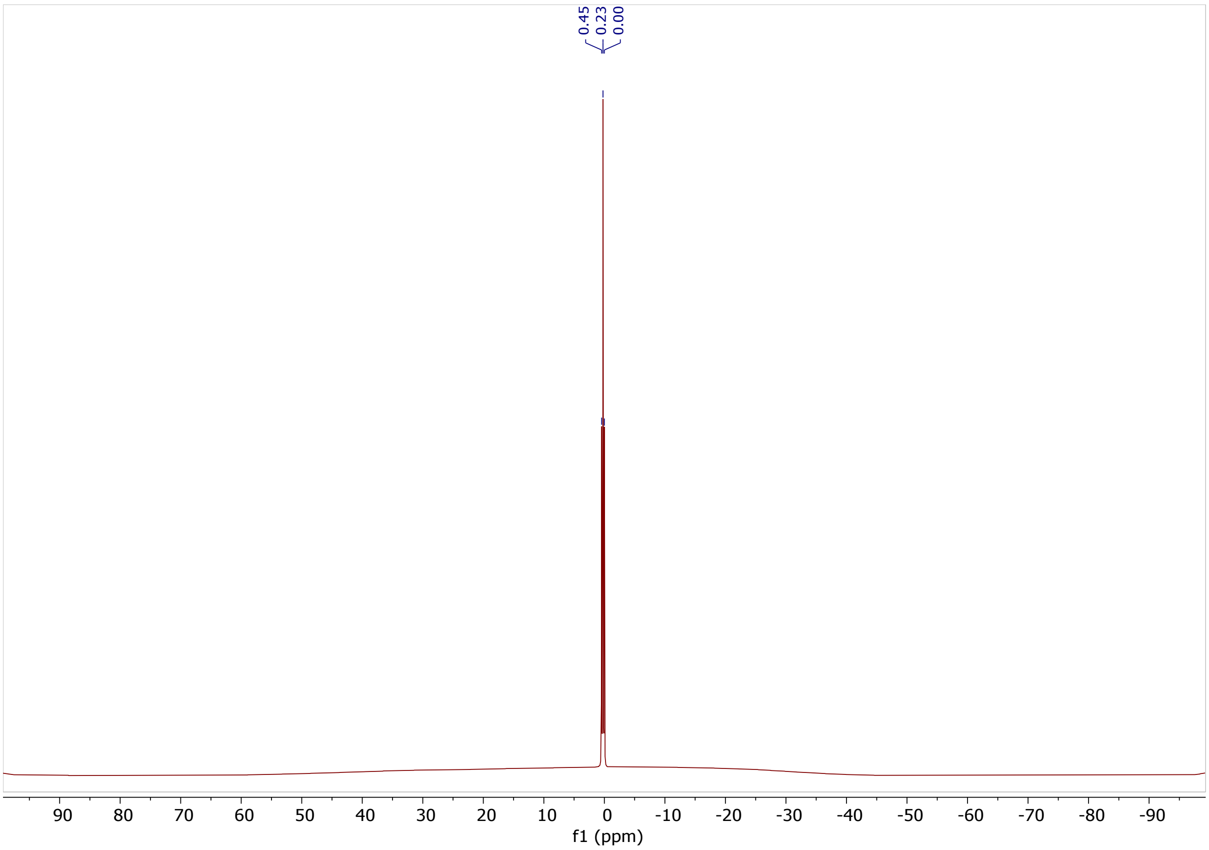

Fig S49. ^{11}B NMR spectrum of **BODIPY-Lys.**

Mass Spectrometry (HPLC-MS) analysis.

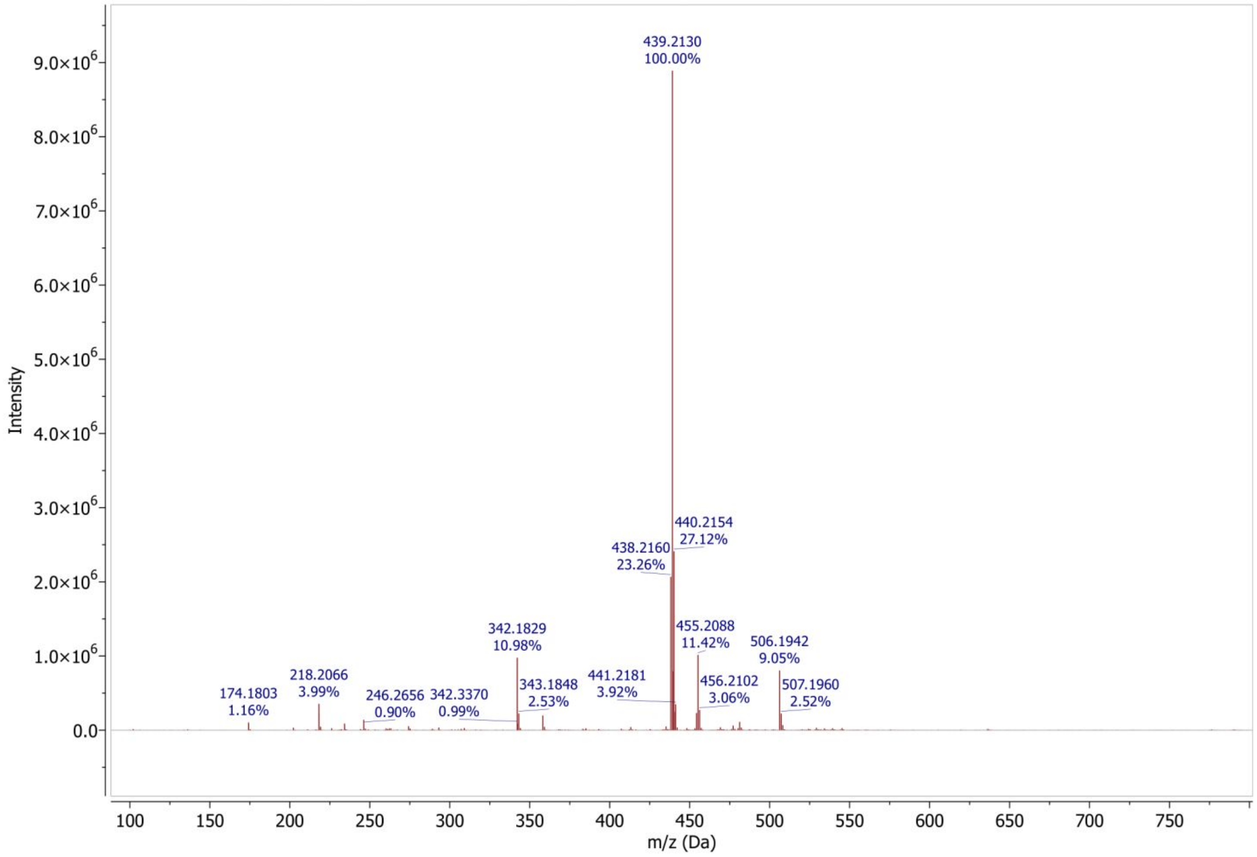

Fig S20. Mass spectrum of **BODIPY-Lys**.

[1] N. Horiuchi, K. Nakagawa, Y. Sasaki, K. Minato, Y. Fujiwara, K. Nezu, Y. Ohe, N. Saijo, In vitro antitumor activity of mitomycin C derivative (RM-49) and new anticancer antibiotics (FK973) against lung cancer cell lines determined by tetrazolium dye (MTT) assay. Cancer Chemother. Pharmacol. 22 (1988) 246–250. https://doi.org/10.1007/BF00273419.

[2] M. Gruzdev, U. Chervonova, N. Bumagina, A. Kolker, Synthesis and Optical Properties of BODIPY with Active Group on meso- Position, Lett. Org. Chem. 13 (2016) 718–725. https://doi.org/10.2174/1570178614666161118155955.

[3] J. Pliquett, S. Amor, M. Ponce-Vargas, M. Laly, C. Racoeur, Y. Rousselin, F. Denat, A. Bettaïeb, P. Fleurat-Lessard, C. Paul, C. Goze, E. Bodio, Design of a multifunctionalizable BODIPY platform for the facile elaboration of a large series of gold(i)-based optical theranostics, Dalt. Trans. 47 (2018) 11203–11218. https://doi.org/10.1039/c8dt02364f.
